# Phylogenomic approaches untangle early divergences and complex diversifications of the olive plant family

**DOI:** 10.1186/s12915-022-01297-0

**Published:** 2022-04-25

**Authors:** Wenpan Dong, Enze Li, Yanlei Liu, Chao Xu, Yushuang Wang, Kangjia Liu, Xingyong Cui, Jiahui Sun, Zhili Suo, Zhixiang Zhang, Jun Wen, Shiliang Zhou

**Affiliations:** 1 Laboratory of Systematic Evolution and Biogeography of Woody Plants, School of Ecology and Nature Conservation, Beijing Forestry University, Beijing, 100083 China; 2 State Key Laboratory of Systematic and Evolutionary Botany, Institute of Botany, Chinese Academy of Sciences, Beijing, 100093 China; 3 State Key Laboratory Breeding Base of Dao-di Herbs, National Resource Center for Chinese Materia Medica, China Academy of Chinese Medical Sciences, Beijing, 100700 China; 4 Department of Botany, National Museum of Natural History, Smithsonian Institution, Washington, DC 20013-7012 USA

**Keywords:** Ancient introgression, Gene tree conflict, Incomplete lineage sorting, Oleaceae, Phylogenomics, Rate heterogeneity

## Abstract

**Background:**

Deep-branching phylogenetic relationships are often difficult to resolve because phylogenetic signals are obscured by the long history and complexity of evolutionary processes, such as ancient introgression/hybridization, polyploidization, and incomplete lineage sorting (ILS). Phylogenomics has been effective in providing information for resolving both deep- and shallow-scale relationships across all branches of the tree of life. The olive family (Oleaceae) is composed of 25 genera classified into five tribes with tribe Oleeae consisting of four subtribes. Previous phylogenetic analyses showed that ILS and/or hybridization led to phylogenetic incongruence in the family. It was essential to distinguish phylogenetic signal conflicts, and explore mechanisms for the uncertainties concerning relationships of the olive family, especially at the deep-branching nodes.

**Results:**

We used the whole plastid genome and nuclear single nucleotide polymorphism (SNP) data to infer the phylogenetic relationships and to assess the variation and rates among the main clades of the olive family. We also used 2608 and 1865 orthologous nuclear genes to infer the deep-branching relationships among tribes of Oleaceae and subtribes of tribe Oleeae, respectively. Concatenated and coalescence trees based on the plastid genome, nuclear SNPs and multiple nuclear genes suggest events of ILS and/or ancient introgression during the diversification of Oleaceae. Additionally, there was extreme heterogeneity in the substitution rates across the tribes. Furthermore, our results supported that introgression/hybridization, rather than ILS, is the main factor for phylogenetic discordance among the five tribes of Oleaceae. The tribe Oleeae is supported to have originated via ancient hybridization and polyploidy, and its most likely parentages are the ancestral lineage of Jasmineae or its sister group, which is a “ghost lineage,” and Forsythieae. However, ILS and ancient introgression are mainly responsible for the phylogenetic discordance among the four subtribes of tribe Oleeae.

**Conclusions:**

This study showcases that using multiple sequence datasets (plastid genomes, nuclear SNPs and thousands of nuclear genes) and diverse phylogenomic methods such as data partition, heterogeneous models, quantifying introgression via branch lengths (QuIBL) analysis, and species network analysis can facilitate untangling long and complex evolutionary processes of ancient introgression, paleopolyploidization, and ILS.

**Supplementary Information:**

The online version contains supplementary material available at 10.1186/s12915-022-01297-0.

## Background

Understanding the evolutionary processes remains central to addressing questions about diversification of life on Earth. One of the most difficult challenges in systematics and evolution is inferring the deep-branching relationships during periods of incomplete lineage sorting (ILS), ancient introgression/hybridization, polyploidization, and rapid radiation. Phylogenomic studies often focus on resolving deep-branching relationships, such as the root of angiosperms [1, 2], the backbone of animals [3], the family relationships of asterids [4], the subfamilies of legumes [5, 6], and deep recalcitrant relationships within a family [7, 8]. These studies have shown that such relationships may remain unresolved even when large genome-scale molecular sequencing data are used, due to the discordant phylogenetic signals among genes from different genomes (nuclear, plastid and mitochondrial genomes) or different genomic regions [9–11]. However, phylogenomic analyses can provide effective information to gain insights into the complexity of evolutionary processes and the underlying causes of the lack of phylogenetic resolution and conflicting phylogenetic results.

One of the most significant phenomena in phylogenomic analyses is gene tree and species tree discordance in empirical studies. Gene tree discordance has numerous causes, such as substitution rate variation [12], gene duplication/loss, gene tree estimation errors, or random noise from uninformative genes [13], as well as ILS and introgression/hybridization [11, 14–17]. Among these potential sources of gene tree discordance, ILS is recognized as the cause to explain conflicting genealogies [17]. ILS or deep coalescence describes the pattern due to stochasticity of the coalescent, representing the retention of ancestral polymorphism and fixation in the descendant lineages after speciation events due to stochastic genetic drift. Meanwhile, introgression/hybridization can similarly result in gene tree discordance. More recently, several methods have been developed to differentiate between the two or infer phylogenetic networks while accounting for ILS and introgression/hybridization simultaneously [18–20], but they are most commonly used at shallow phylogenetic scales, such as the species level [21–23]. For deeper phylogenetic scales (such as at the subfamily level or genus level), distinguishing true discordance causes can be challenging because the long history of evolutionary processes may obscure phylogenetic signals [6, 24, 25]. To overcome these limitations, comparing phylogenetic signals among genetic markers with different inheritances (plastid and nuclear genomes) and the use of multiple phylogenetic tools are essential to disentangle causes of phylogenetic conflict and provide insight into evolutionary histories.

The olive family (Oleaceae) is composed of 25 genera and approximately 600 species of temperate and tropical shrubs or woody climbers and trees distributed from the north temperate to the southern parts of Australia, Africa, and South America. Oleaceae are important components of temperate and tropical ecosystems [26, 27]. Moreover, many Oleaceae species are economically important, e.g., olive (*Olea europaea*) is cultivated for its fruit and oil, *Jasminum*, *Forsythia*, *Osmanthus*, *Syringa*, and *Ligustrum* are cultivated extensively as ornamentals and for fragrances, and ash trees (*Fraxinus*) are grown for timber as well as ornamentals.

Within the Lamiales, Oleaceae is sister to the small tropical Asian family Carlemanniaceae, and the clade is the early divergent group in Lamiales [4, 28]. More than two decades since the first molecular phylogenies of the Oleaceae were inferred [26], the family has now been supported to include five tribes (Myxopyreae, Fontanesieae, Forsythieae, Jasmineae, and Oleeae), and the tribe Oleeae is divided into four subtribes (Schreberinae, Ligustrinae, Fraxininae, and Oleinae). The evolutionary history of Oleaceae is very complex, e.g., Oleeae originated from paleopolyploid events with one of the parental genome closely related to *Jasminum* [29], and some of the recognized genera are polyphyletic [26, 30–37] or paraphyletic [38]. Furthermore, phylogenetic incongruence between plastid and nuclear data has been reported, suggesting ILS and/or hybridization within several genera [34, 39]. Heterogeneous evolutionary rates among clades and genes might also account for conflicting relationships [35–37].

**Fig. 1:**
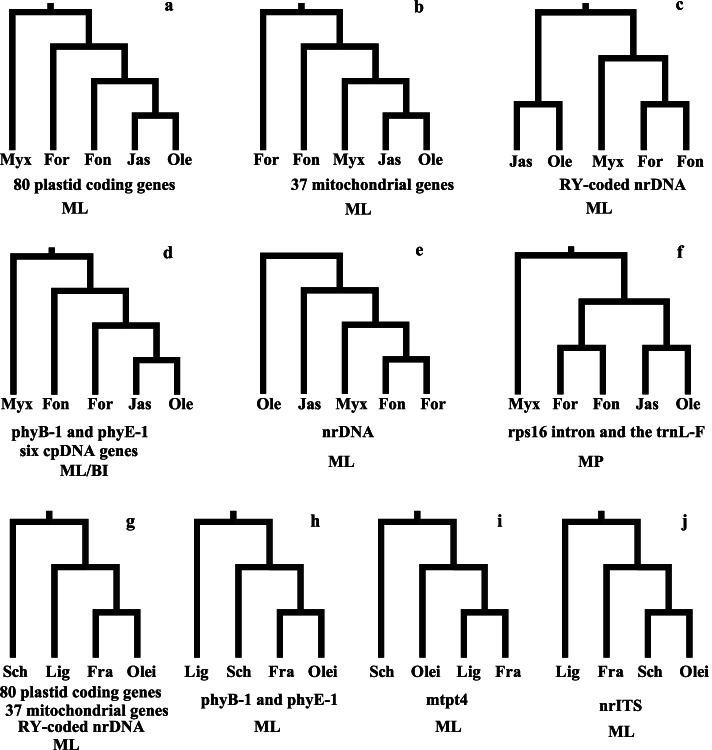
Phylogenetic hypotheses of Oleaceae from previous studies. **a–f** The six alternate topologies of the five tribes. **g–j** The four alternate topologies of the four subtribes of Oleeae. **a** Dupin et al. [36] using the 80 concatenated plastid coding genes based on the maximum likelihood (ML) method. **b** Dupin et al. [36] using the 37 concatenated mitochondrial genes based on the ML method. **c** Dupin et al. [36] using the RY-coded nrDNA based on the ML method. **d** Ha et al. [40] using six cpDNA sequence datasets (*matK*, *rbcL*, *ndhF*, *atpB*, *rps16*, and *trnL-F*) based on the Bayesian inference (BI) method and Dupin et al. [36] using the nuclear genes of phyB-1 and phyE-1. **e** Dupin et al. [36] using the nontransformed nrDNA cluster based on the ML method. **f** Wallander and Albert [26] using two plastid genes, *rps16* and *trnL-F*, based on maximum parsimony (MP) methods. **g** Dupin et al. [36] using the 80 concatenated plastid coding genes, 37 concatenated mitochondrial genes, and RY-coded nrDNA based on the ML method. **h** Dupin et al. [36] using the nuclear genes of phyB-1 and phyE-1. **i** Van de Paer et al. [41] using the nuclear mtpt4 based on the ML method. **j** Dupin et al. [36] using the nontransformed nrDNA cluster based on the ML method. Myx, Myxopyreae; Fon, Fontanesieae; For, Forsythieae; Jas, Jasmineae; Ole, Oleeae; Lig, Ligustrinae; Sch, Schreberinae; Fra, Fraxininae; Olei, Oleinae

Previous molecular phylogenetic analyses did not well resolve the origin and early evolution, including deep-branching relationships among the five tribes and subtribes of Oleeae (Fig. 1). Six and four possible topologies among five tribes and four subtribes of Oleeae, respectively, appeared in previous studies and showed obvious incongruence when using datasets from different genomes. Moreover, previous olive phylogenies have been heavily relied on chloroplast and mitochondrial markers [39, 41], and a handful of nuclear genes have shown different topologies [36]. Extensive sampling of molecular datasets, especially unlinked nuclear genes, which can account for different evolutionary histories of individual genes, is preferable to infer species trees and explore the causes of conflicts for deep branching.

Beyond resolving the complex history in the olive family, our main objectives are to investigate the causes of the lack of resolution, distinguish phylogenetic signal conflicts, and explore alternative scenarios for the uncertainties concerning deep-branching relationships of the olive family. First, we estimated the olive family relationships using 180 samples from 24 genera representing all five tribes based on the whole plastid genomes and nuclear SNP datasets. These analyses were used to test whether the markers of different inheritance caused the lack and/or conflict of phylogenetic signals. We employed multiple phylogenetic methods and data partitioning schemes to resolve recalcitrant relationships at both deep and shallow nodes. Second, we analyzed thousands of nuclear gene alignments harvested from whole genome sequencing and published complete genomes of representative species from the tribes or subtribe of Oleeae. Upon inferring the most likely species tree, we analyzed and distinguished the signal of gene tree discordance produced by ILS, introgression/hybridization, and hard polytomy among deep branches and explored the implications for understanding the early evolutionary diversification of the olive family.

## Results

### Phylogenomic relationships based on plastid datasets and molecular evolutionary rate variation among clades of Oleaceae

**Fig. 2:**
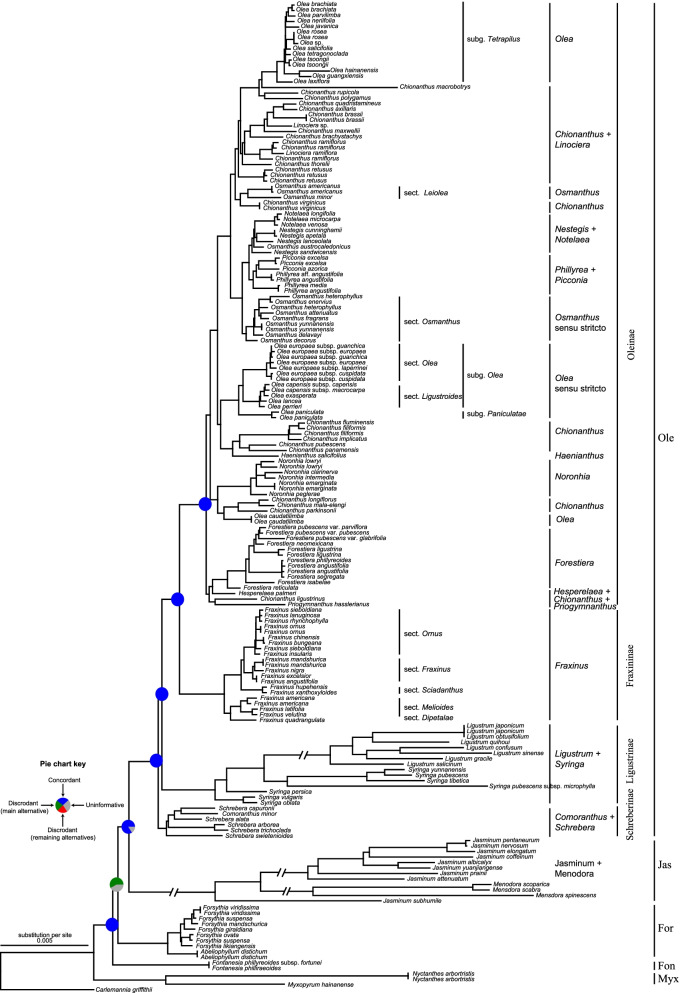
Maximum likelihood phylogeny of Oleaceae inferred from RAxML analysis of the plastid 77G180saa dataset based on the gene partition models. Pie charts present the proportion of 19 plastid gene trees that support that clade (blue), or support the main alternative bifurcation (green), or support the remaining alternative (red), and the proportion that have < 80% bootstrap support (gray). Only pie charts for major clades are shown, and Additional file 2: Fig. S1 shows pie charts for all nodes. Myx, Myxopyreae; Fon, Fontanesieae; For, Forsythieae; Jas, Jasmineae; Ole, Oleeae

To resolve the phylogeny of Oleaceae, we expanded the taxon sampling (Additional file 1: Tables S1-S2), employed extensive data from plastid genomes, and used multiple methods to dissect the phylogenetic signals (Table 1 and Table 2), and explore information and conflicts among the phylogenetic trees. In total, seven plastid datasets were constructed to infer the phylogeny of Oleaceae (Table 1), and a total of 19 ML (maximum likelihood) trees (Table 2) were constructed based on different datasets and phylogenetic methods. The ML tree from the 180s77Gaa dataset under a gene partitioning scheme was used as our main reference or summary tree for iterative topological concordance analyses of the plastid gene trees (Fig. 2, Table 2, Additional file 2: Fig. S1, and the reason for using this tree as the reference tree was shown in Additional file 3), which visualized the proportions of genes in each gene tree supporting the alternative topologies. Our analyses revealed all tribes as monophyletic with full support. However, conflicting topologies were detected at several nodes among different trees (see below). The relationships among the tribes were less robustly resolved, with, in particular, the positions of Fontanesieae and Forsythieae showed conflicts in some analyses (Fig. 2). Myxopyreae, the first diverged lineage of the olive family, was strongly supported in all analyses. The plastid nucleotide sequence datasets and the 180s77Gaa based on the posterior mean site frequency (PMSF) model supported Forsythieae as sister to the clade comprising Fontanesieae, Jasmineae, and Oleeae (topology **a** (Myxopyreae (Forsythieae, (Fontanesieae, (Jasmineae, Oleeae))) in Fig. 1). In contrast to the plastid nucleotide sequence phylogeny, the analyses of the amino acid sequence data (180s77Gaa) except under the PMSF model showed that Fontanesieae was sister to the clade comprising Forsythieae, Jasmineae, and Oleeae (topology **d** (Myxopyreae (Fontanesieae, (Forsythieae, (Jasmineae, Oleeae))) in Fig. 1). However, this topology was weakly supported by the 180s77Gaa and the bootstrap support values were 25%, 32%, and 35% using the three partitioning schemes (Table 3 and Additional file 1: Table S3). This suggests that the topology **a** of the five tribes in Fig. 1 is the most likely, as inferred from the plastid data, with the high support values when using the whole plastome data. The phylogenetic signal in the plastid data with regard to this topology appears to be sufficient. The sister relationship of Jasmineae and Oleeae was strongly supported in all analyses.

**Table 1:** Characteristics of data matrices of plastomes and SNP data

Datasets	Description	Number of sequences	Alignment length (bp)	Variable sites		Information sites	
				Numbers	%	Numbers	%
CPG-complete	Complete plastome data	180	152,399	32,599	21.39	22,461	14.74
CPG-trimAl-automated1	Complete plastome data trimmed by TrimAI using atuomated1 method	180	126,241	29,945	23.72	21,324	16.89
CPG-trimAl-nogaps	Complete plastome data trimmed by TrimAI using nogaps method	180	83,647	16,189	19.35	11,370	13.59
CPG-trimAl-strict	Complete plastome data trimmed by TrimAI using strict method	180	114,523	20,327	17.75	13,434	11.73
CPG-trimAl-strictplus	Complete plastome data trimmed by TrimAI using strictplus method	180	108,581	18,774	17.29	12,477	11.49
180s77Gnt	The nucleotide sequences of all protein coding loci including all taxa	180	55,296	10,120	18.30	7047	12.74
180s77Gaa	The amino acid sequences of all protein coding loci including all taxa	180	18,742	2841	15.16	1605	8.56
91s77G	A reduced sample set with nearly all representative lineages of Oleaceae	91	55,296	9111	16.48	5605	10.14
SNP-olea	SNPs identified using the *Olea* genome as reference	180	186,053	186,053	100	183,854	98.82
SNP-ash	SNPs identified using the ash genome as reference	180	408,702	408,702	100	401,076	98.13
SNP-suspen	SNPs identified using the *Forsythia suspensa* genome as reference	180	91,534	91,534	100	88,936	97.17

**Table 2:** Summary of the methods used for building gene trees. Twenty-five gene trees were reconstructed based on the 77 plastid coding genes, complete plastome data, and the SNP datasets. The number in the sheet represents each analysis

Program	Site model	Dataset									
		77 plastid coding genes		complete plastome data					SNPs data		
		77 g nt	77 g aa	complete	trimAl_automated1	trimAl-nogaps	trimAl-strict	trimAl-strictplus	SNP-olea	SNP-ashtree	SNP-suspen
RaxML	ModelFinder	Unpartitioned (1)	Unpartitioned (6)	yes (10)	yes (11)	yes (12)	yes (13)	yes (14)	yes (20)	yes (21)	yes (22)
RaxML	N/A	PartitionFinder (2)	PartitionFinder (7)	N/A	N/A	N/A	N/A	N/A	N/A	N/A	N/A
RaxML	N/A	Gene partitioned (3)	Gene partitioned (8)	N/A	N/A	N/A	N/A	N/A	N/A	N/A	N/A
RaxML	N/A	Codon partitioned (4)	N/A	N/A	N/A	N/A	N/A	N/A	N/A	N/A	N/A
IQ-TREE	PMSF	N/A	yes (9)	N/A	N/A	N/A	N/A	N/A	N/A	N/A	N/A
IQ-TREE	GHOST	Yes (5)	N/A	Yes (15)	Yes (16)	Yes (17)	Yes (18)	Yes (19)	Yes (23)	Yes (24)	Yes (25)

**Table 3:** Comparison of partition model from maximum likelihood analysis

Dataset	Partition schemes	No. partitions	2logeL	No. free parameters	AICc
180s77Gnt	Unpartitioned	1	-194843.878	366	390424.65
	PartitionFinder	31	-192293.919	666	85936.101
	Gene partitioned	31	-191926.419	684	385238
	Codon partitioned	3	-191946.283	389	384676.09
180s77Gaa	Unpartitioned	1	-123709.795	357	248147.73
	PartitionFinder	36	-120467.852	428	241811.76
	Gene partitioned	31	-120518.104	418	241891.32

In contrast to the problematic deep relationships of the family, our analyses robustly supported relationships among major clades within the tribe Oleeae. There was 100% support for the monophyly of each subtribe, and the topology of (Schreberinae (Ligustrinae, (Oleinae, Fraxininae))) (topology **g** in Fig. 1) was strongly supported by all the analyses, consistent with previous studies [35, 36]. Within the Oleeae, at least seven genera were not monophyletic (i.e., *Schrebera*, *Syringa*, *Chionanthus*, *Olea*, *Osmanthus*, *Phillyrea*, and *Nestegis*), and *Chionanthus* was the most complex polyphyletic genus (Fig. 2). Three genera including *Forestiera*, *Hesperelaea*, *Priogymnanthus*, and the species *Chionanthus ligustrinus* formed a highly supported clade and were sister to the rest of the subtribe Oleinae. The internode certainty all (ICA) value for the backbone of Oleinae was low (Fig. 2 and Additional file 2: Fig. S1), indicating major incongruence between species trees. The conflict can therefore, at least partially, reflect incomplete sorting and/or introgression/hybridization [33, 35].

**Fig. 3:**
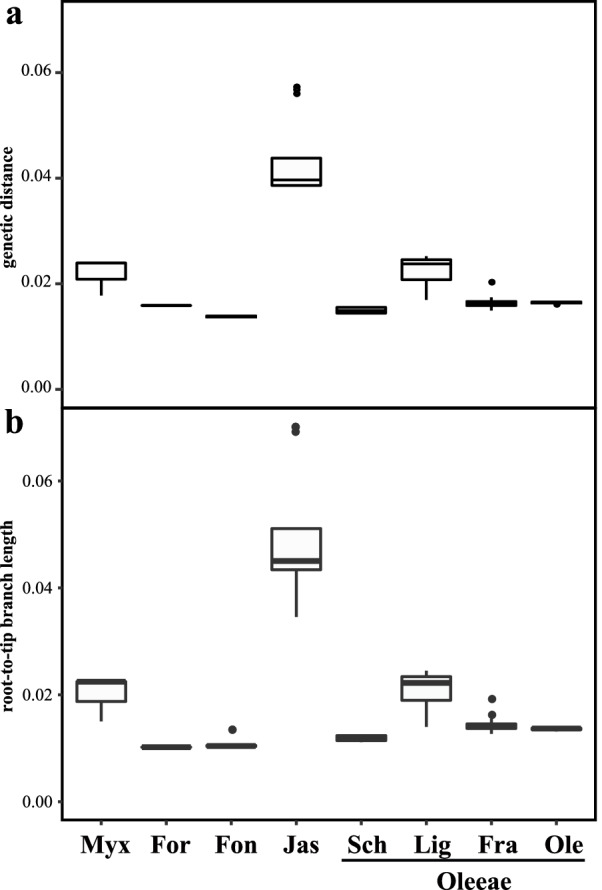
Variation in plastid substitution rates among clades of Oleaceae. **a** Genetic distance among clades/branches of Oleaceae. **b** Comparison of intratribal and intrasubtribal plastid branch lengths among the Oleaceae based on the ML tree of the “77G180snt” dataset using the gene partitioned model, as assessed by root-to-tip branch lengths, from the common ancestor of each respective clade to each sampled tip

The ML tree based on the plastid genome data showed significant differences in branch lengths (Fig. 2 and Fig. 3b) among the tribes and subtribes of Oleaceae. The tribe Jasmineae and the Oleeae subtribe Ligustrinae had the longest branch lengths, while Forsythieae and Oleeae had relatively short branch lengths. Genetic distances showed a similar pattern with branch lengths (Fig. 3a).

Branch model tests in Baseml/PAML indicated that the results significantly departed from the null hypothesis that all rates were equal among clades (“global clock” model) (Table 4). Model M1, which allows a local clock for Jasmineae, had a significantly better fit than M0. The rates for Jasmineae branches were 5.58 times higher than the background (Table 4). Meanwhile, Model M2 (a local clock for Jasmineae and the Oleeae subtribe Ligustrinae) had a better fit than Model M1, and the rates for Jasmineae and the Oleeae subtribe Ligustrinae were 6.98 and 2.29 times higher than those for the remaining Oleaceae species. According to the AICc comparison and Bonferroni-corrected likelihood ratio tests, Model M3 was the best fitting model, which indicated that Oleaceae had branch rate variation among the most clades.

**Table 4:** Model comparisons of global vs local clocks using the baseml module of PAML

Model	M0	M1	M2	M3
Description	Global Clock	Jasmineae (local) vs M0	Jasmineae and Ligustrinae (each local) vs M1	Jasmineae, Oleeae, Forsythieae, and Ligustrinae (each local) vs M2
No. parameters	188	189	190	192
No. branch parameters	0	1	2	4
AICc	397616.0097	393910.5308	393526.1902	393391.5477
—log_e_L	198619.36	196765.6138	196572.4365	196503.1014
Λ	Na	3707.492572	386.354476	138.670312
df	Na	1	1	2
*P*-value/ Bonferroni corrected	Na	***/***	***/***	***/***
Clade rate parameters (relative	Na	Jas = 5.58123	Jas = 6.98256	Jas = 4.90304
to background rate of 1)			Lig = 2.29322	Lig = 1.61560
				Ole = 0.70533
				For = 0.42790

No. parameters, the total number of free parameters for a particular model

No. branch parameters, number of clades defined to evolve by a local clock

—log_e_L, the likelihood of the data, given the model

AICc, corrected Akaike Information, AICc = -2(log_e_*L*) + 2*K*(*n*/(*n*-*K*-1)) and log_e_L is the likelihood function, *n* is the number of sites in the alignment, and *K* is the number of free model parameters

Λ, the chi-square distributed, Λ = 2 * the difference in log_e_L between two models

df, degree of freedom, df = the difference in no. parameters between two models

*P* value, significance of log likelihood ratio test comparing fit of two models (including after Bonferroni correction where *P* = *α*/*m*, and *m* is the number of branch parameters)

Na, not applicable

***P < 0.001; Jas = Jasmineae; Lig = Ligustrinae; Ole = Oleeae; For = Forsythieae

### Phylogenomic relationships of Oleaceae based on nuclear datasets

**Fig. 4:**
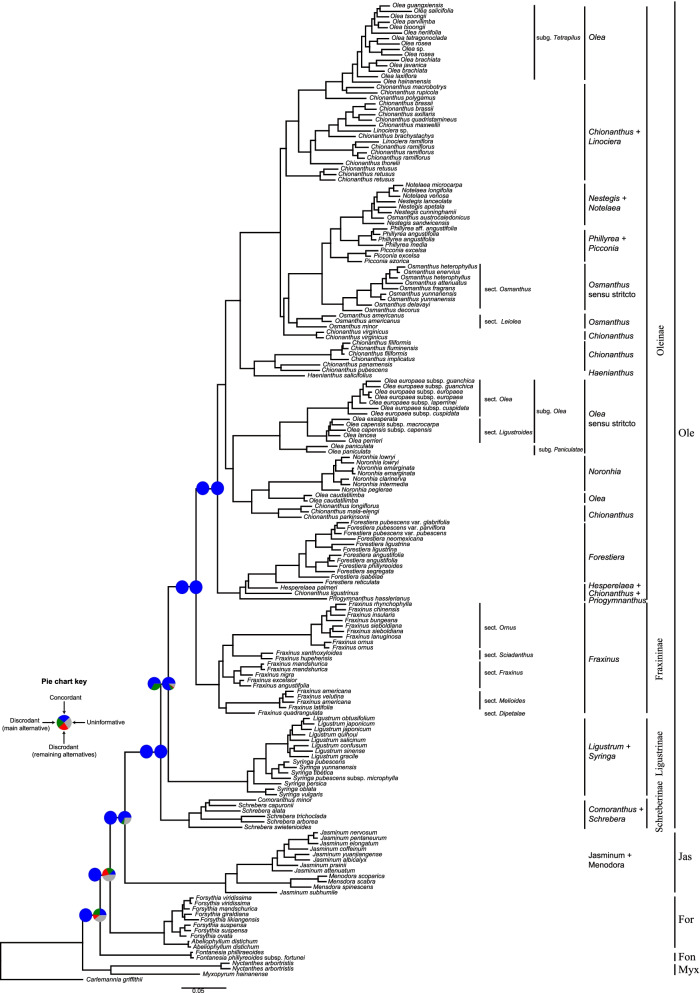
Maximum likelihood phylogeny of Oleaceae inferred from RAxML analysis of the SNP-ash dataset. The left and the right pie charts presented the proportion of nine SNP data trees and the proportion of 41 gene trees based on the dividing method using the SNP-ash dataset, respectively. The pie charts indicate support for that clade (blue), or support for the main alternative bifurcation (green), or support for the remaining alternative (red), and the proportion that have < 80% bootstrap support (gray). Only pie charts for major clades are shown, and Additional file 2: Fig. S2 and S3 shows pie charts for all nodes. Myx, Myxopyreae; Fon, Fontanesieae; For, Forsythieae; Jas, Jasmineae; Ole, Oleeae

Following the methods of Olofsson et al. [35], we obtained three nuclear SNP datasets using the oleaster (*Olea europaea* var. *sylvestris*), ash (*Fraxinus excelsior*), and *Forsythia suspensa* nuclear genomes as the reference sequences (Table 1). Finally, six gene trees were reconstructed using two phylogenetic methods (Table 2). Using the SNP-ash dataset, 41 gene trees were reconstructed. These results were showed in Fig. 4, Additional file 2: Fig. S2 and Fig. S3, respectively.

All six gene trees from the three SNP datasets supported that all tribes and subtribes of Oleeae were monophyletic groups, with concordant relationships among the deep nodes in the six trees (Fig. 4 and Additional file 2: Fig. S2). The topology of (Myxopyreae (Fontanesieae, (Forsythieae, (Jasmineae, Oleeae))) (topology **d** in Fig. 1) for the five tribes of Oleaceae and the topology of (Schreberinae (Ligustrinae, (Oleinae, Fraxininae))) (topology **j** in Fig. 1) for the four subtribes of Oleeae were strongly supported. The nuclear SNP datasets also inferred that seven genera were not monophyletic in Oleeae. Most of the backbone of Oleinae were resolved with high ICA values. Furthermore, some nodes had major conflicts among the gene trees, such as the backbone of *Fraxinus* (Additional file 2: Fig. S2)*.*

At the tribe level, the backbone relationships had low support and showed conflicting phylogenetic signals (Fig. 4) using the SNP dataset, indicating a complex early evolutionary history. The four subtribes of Oleeae were well supported, consistent with whole SNP dataset results. The SNP dataset suggested that some shallow nodes had conflicting phylogenetic signals, e.g. the species relationship among *Ligustrum*, and *Olea* (Additional file 2: Fig. S3).

### Assessing phylogenetic relationships and conflicts of phylogenetic signals

Half of the nodes had a consistent topology among the 25 gene trees (plastid and nuclear SNP dataset, Additional file 2: Fig. S4); however, the backbone of the family was characterized by high levels of gene tree discordance. The most significant conflicting nodes are at the tribe level, and our data supported two alternative topologies (topology **a** and **d** in Fig. 1). The incongruence was higher at the shallow branches, but generally, most conflicting nodes had a majority uninformative gene tree (Additional file 2: Fig. S4). For example, most trees (17/25) were uninformative at the node of the sister group relationship between *Olea javanica* and the clade consisting of *O. neriifolia*, *O. parvilimba*, and *O. brachiata*. Insufficient information could lead to spurious tree inference, thus producing noise and/or conflict.

**Fig. 5:**
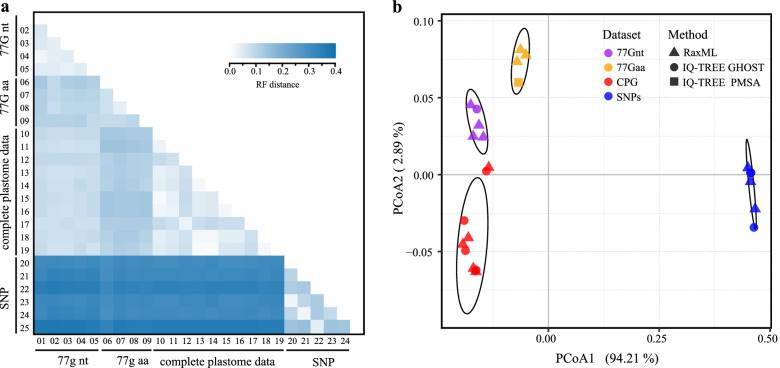
Comparison of topologies of multiple gene trees. Twenty-five gene trees were reconstructed based on the 77 plastid coding genes, plastome data, and SNP datasets. **a** Matrix of Robinson-Foulds (RF) distance, which measures the overall topological discrepancy between two trees. The numbers in the *x*-axis and *y*-axis represented the gene trees, and the information was showed in Table 2. **b** PCoA of the RF distance matrix

Overall, the three types of datasets showed incongruence in topology when compared with trees derived from implicit (e.g., distance-based) analyses (Fig. 5a). The nuclear SNP trees, in particular, had high support values in the backbone branches. This high resolution is directly related to the larger sampling of parsimony-informative sites (Table 1). On the other hand, the phylogenetic relationships recovered by the plastid data were impacted by the robustness of the method. Meanwhile, the nuclear SNPs sampled across the genome are probably unlinked, while the plastid genes constitute just a single locus. These two types of datasets hence track different evolutionary histories, leading to the incongruence in topology.

To further evaluate the impact of heterogeneity of sequence evolution across sites on relationships, we used the heterogeneous model, PMSF, and general heterogeneous evolution on a single topology (GHOST), which considers heterogeneity in the amino acid and nucleotide substitution process. The impact of using the GHOST model instead of homogeneous models on the topology was small compared with the data types (Fig. 5a). Meanwhile, the GHOST and PMSF trees continued to support a large portion of phylogenetic relationships among the deep nodes. The PMSF trees have different topologies (topology **a** in Fig. 1) among the five tribes compared to the trees from site homogeneous models (topology d in Fig. 1). Gene partitioned analyses using the two plastid gene datasets (180s77Gnt and 180s77Gaa) also produced fewer effective topologies.

The principal coordinates analysis (PCoA) (Fig. 5b) showed that all nuclear SNP gene trees were clearly separated from the plastid gene trees along the first and the second axes. The three plastid gene trees were separated along the second axis. Within the datasets, gene trees obtained with different phylogenetic methods are spread across the tree space.

### Widespread introgression across the five tribes in Oleaceae

**Fig. 6:**
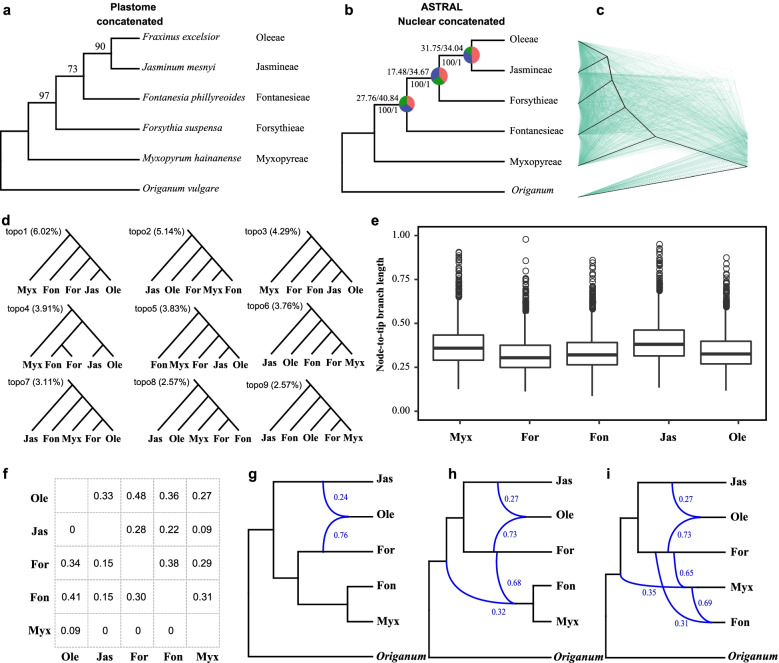
Phylogeny and tests for gene introgression of five tribes of Oleaceae. **a** Plastome concatenated tree inferred from a 76-coding gene supermatrix. **b** ASTRAL species tree and the nuclear concatenated phylogeny inferred from 2608 nuclear genes. Pie charts in the nodes present the proportion of gene trees that support the main topology (red), the first alternative (blue), and the second alternative (green). Gene concordance factor (gCF)/site concordance factor (sCF) values are shown above the branches. ML bootstrap/astral local posterior probabilities are shown below branches. **c** Cladograms of the coalescent-based species tree (heavy black lines) and 500 gene trees (in green) randomly sampled from 2608 inferred gene trees. **d** The most common topologies in gene trees, sorted by frequency of occurrence, as shown in brackets. **e** Comparison of branch length of five tribes. The root-to-tip branch length of each gene tree and each sample were assessed. **f** Pairwise *D* per species pair (lower diagonal) and the mean total proportion of introgressed loci per species pair inferred through QuIBL analysis (upper diagonal). 0 values correspond to nonsignificant values. More details were provided in Table S5. **g**–**i** Phylogenetic network analysis using PhyloNet. Numerical values next to curved branches indicate inheritance probabilities for each hybrid node. Myx, Myxopyreae; Fon, Fontanesieae; For, Forsythieae; Jas, Jasmineae; Ole, Oleeae

To further assess inherent conflicts between gene trees and species trees across the five tribes in Oleaceae, we estimated the plastid genome tree, individual nuclear gene trees and a species tree based on the 2608 single-copy orthologous genes among the five species representing the five tribes and the outgroup *Origanum vulgare* (Fig. 6a, b)*.* The plastid genome tree showed that Fontanesieae was sister to a clade of Jasmineae and Oleeae, while there was inconsistency with the species tree, and the nuclear concatenated gene tree, which supported Forsythieae, Jasmineae, and Oleeae forming a clade. All branches in the species tree had low major quartet scores (q1), gene concordance factor (gCF), and site concordance factor (sCF) of < 0.5 (Fig. 6b), and these three branches received almost equal quartet scores for q1, q2, and q3, suggesting that the gene trees yielded random topologies with respect to the species tree, which was also supported by the overlapping gene trees (Fig. 6c).

All the frequencies of 105 possible topologies were shown in Additional file 1: Table S4, and 103 possible topologies appeared in the 2608 gene trees. The number of the eleven most frequent topologies (topo1 to topo9) ranged from 6.02% to 2.57% (Fig. 6d), indicating significant conflict among the gene trees. Only 6.02% of these gene trees (topo1) were consistent with the species tree, and the plastid genome tree (topo3) was the third most frequent topology, accounting for 4.29%. The second most frequent topologies (topo2, accounting for 5.14%) showed that Jasmineae and Oleeae were the first and second divergent groups, respectively, and Forsythieae was sister to a clade of Myxopyreae and Fontanesieae. One-way analysis of variance test showed the branch lengths of all gene trees among the five nodes had significant differences (*P* < 0.05), indicating that there was rate variation among the tribes in the nuclear data (Fig. 6e). The ASTRAL polytomy tests resulted in the same bifurcating species tree for the nuclear gene dataset and rejected the null hypothesis that any branch was a polytomy (*P* < 0.01).

To further assess whether the observed gene tree incongruences were mainly due to hybridization/gene flow, we calculated the *D*-statistic, which uses the ABBA-BABA test for introgression between species. The *D*-statistic showed that *D* was significant in all the triplets (*P* < 0.002, *Z* > 3; Additional file 1: Table S5). A mean value of absolute *D* for a species pair was calculated from all triplets (Fig. 6f and Additional file 1: Table S5). The absolute *D* was significant in most of the pairwise species comparisons (six out of ten pairwise comparisons) and varied from 0.09 to 0.41 (Fig. 6f). The highest *D* value was among Forsythieae, Oleeae, and Fontanesieae, which could explain the phylogenetic relationships of topo4, topo7, topo8, and topo11 in which Fontanesieae was sister to Forsythieae or Oleeae. For Oleeae and Jasmineae, *D* was not significantly different from zero, and Myxopyreae showed little or no gene flow with the other four tribes. Considering the lower support value and the *D* value of the five tribes, gene flow might have contributed to the observed phylogenetic discordance.

Phylogenetic incongruences can be potentially associated with both ILS and introgression, and the quartet scores (QS) values for q1, q2, and q3 were almost equal, indicating a high level of ILS [42]. We used a recently developed tree-based method, QulBL [19], to distinguish these two processes. The QulBL analysis revealed that most of the triplets showed significant evidence for introgression (26 of 30 triplets, dBIC < − 10, Additional file 1: Table S6). The mean value of the proportion of trees arising via introgression for a species pair was calculated from all triplets (Additional file 1: Table S7). We found a strong signal for gene flow among all ten species pairs (Fig. 6f), suggesting widespread introgression across the ancestral region of the five tribes.

Furthermore, we inferred the phylogenetic networks to visualize gene flow among the five tribes. The PhyloNet analyses identified extremely complicated and statistically significant signals for gene flow across the five tribes (Fig. 6g–i). When reticulation events were set to 1, 2, and 3, all corresponding optimal networks supported the hybrid origin of the tribe Oleeae (*n* = 46) between tribe Forsythieae and tribe Jasmineae. The tribe Oleeae was connected to Forsythieae by an inheritance probability of 0.76, 0.73, and 0.73, respectively, under the three different reticulation scenarios. In each of the three reticulation events, large portions of the genome were exchanged. The other two reticulations are between the ancestral lineage of Jasmineae/Forsythieae/Oleeae (inheritance probability: 0.35) and Myxopyreae (0.65) and between Forsythieae (0.31), and Myxopyreae (0.69). These reticulation events were all supported by the *D*-statistic or QulBL.

Collectively, our results suggested that introgression/hybridization, rather than ILS, was the main factor contributing to the phylogenetic discordance among the five tribes. Oleeae is especially evident with its origin supported by ancient hybridization and polyploidy, with the ancestral lineages of Jasmineae and Forsythieae as the most likely parentages .

### Comparison of genome collinearity between Oleeae and two putative parental tribes

**Fig. 7:**
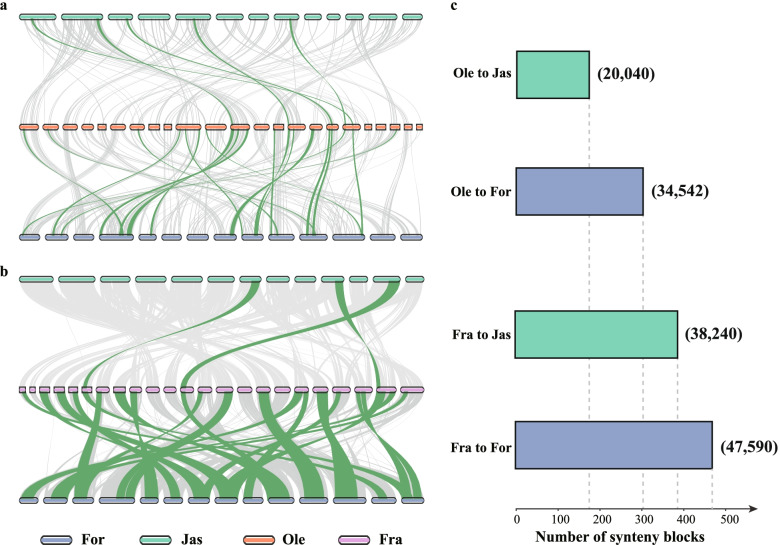
Comparisons of genome synteny of Oleeae with that of Forsythieae and Jasmineae. Two genome synteny plots were generated for *Olea europaea* and *Fraxinus excelsior* of Oleeae with *Jasmimum sambac* and *Forsythia suspensa*, respectively. **a** Synteny of *Olea europaea* with the putative parental lineages: there were 303 synteny blocks found with *Forthysia suspensa* while there were 173 synteny blocks found with *Jasmimum sambac.* **b** Synteny of *Fraxinus excelsior* with the putative parental lineages: there were 470 synteny blocks found with *Forsythia suspensa* while there were 388 synteny blocks found with *Jasmimum sambac*. Top 5% of most similar syntenic blocks’ ribbons were marked as green. **c** Bar plot of numbers of synteny blocks from different synteny combinations. The numbers in parentheses represent the number of syntenic sequences. For, *Forsythia suspensa*.; Jas, *Jasmimum sambac*; Ole, *Olea europaea*; Fra, *F. excelsior*

In order to further identify the parentages of tribe Oleeae, we compared the genome collinearity among Oleeae, Jasmineae, and Forsythieae (Fig. 7). After the BLAST searches, for transcripts of *O. europaea*, there were 20,040 sequences that were successfully mapped to the genome of *J. sambac* while 34,542 sequences were mapped to the genome of *Forthysia suspensa*. For transcripts of *Fraxinus excelsior*, there were 38,240 sequences that were mapped to the genome of *J. sambac*, while 47,590 for *Forthysia suspensa*. The genome synteny comparison of *O. europaea* and *Fraxinus excelsior* with their putative parental lineages showed that there were 173 synteny blocks found between genomes of *O. europaea* and *J. sambac*, fewer than the synteny blocks between *O. europaea* and *Forthysia suspensa* (303)*.* The same result was found in comparisons between *Fraxinus excelsior* and the putative parent lineages: 388 synteny blocks with *J. sambac* and 470 synteny blocks with *Forthysia suspensa* (Fig. 7)*.* Hence, the two gene copies in Oleeae from the putative ancestral lineages (Jasmineae and Forsythieae) showed unequal inheritance. Alternatively, Jasmineae may not be the direct parental lineage.

### ILS and introgression as the main sources of phylogenetic discordance of the four subtribes in tribe Oleeae

**Fig. 8:**
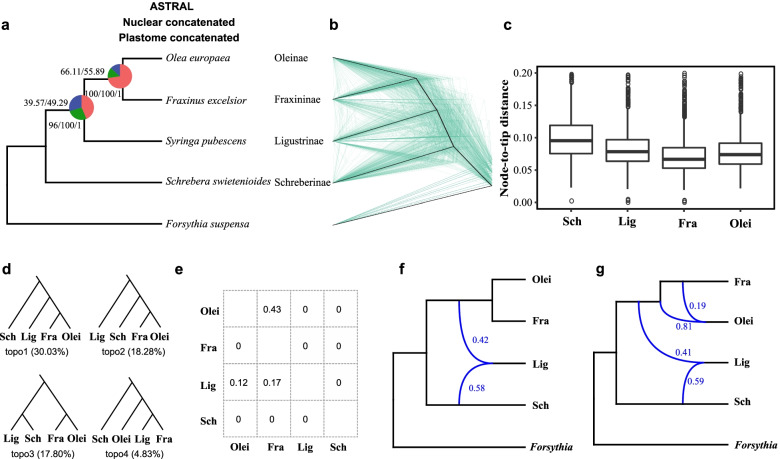
Phylogeny and tests for gene introgression of four subtribes of Oleeae. **a** Plastome concatenated tree inferred from 76-coding gene supermatrix, ASTRAL species tree and the nuclear concatenated phylogeny inferred from 1865 nuclear genes. Pie charts in the nodes present the proportion of gene trees that support the main topology (red), the first alternative (blue), and the second alternative (green). Gene concordance factor (gCF)/site concordance factor (sCF) values are shown above the branches. ML bootstrapping with chloroplast genes and nuclear genes and astral local posterior probability are shown below branches. **b** Cladograms of the coalescent-based species tree (heavy black lines) and 500 gene trees (in green) randomly sampled from 1,865 inferred gene trees. **c** Comparison of branch length of four subtribes. The root-to-tip branch length of each gene tree and each sample were assessed. **d** The most common topologies in gene trees, sorted by frequency of occurrence, as shown in brackets. **e** Pairwise *D* per species pair (lower diagonal) and the mean total proportion of introgressed loci per species pair inferred through QuIBL analysis (upper diagonal). 0 values correspond to nonsignificant values. More details were provided in Table S9. **f**, **g** Phylogenetic network analysis using PhyloNet. Numerical values next to curved branches indicate inheritance probabilities for each hybrid node. Lig, Ligustrinae; Sch, Schreberinae; Fra, Fraxininae; Olei, Oleinae

The plastid genome data, nuclear concatenated gene tree, and species tree based on 1865 single-copy orthologous genes had identical topologies, supporting Schreberinae as the first divergent group, and Ligustrinae forming a clade with Oleinae and Fraxininae. Gene tree concordance factors (QS, gCF, and sCF) showed that the nodes of the clades of Ligustrinae, Fraxininae, and Oleinae were supported by only small fractions, and the QS, gCF, and sCF values were 0.44, 39.57, and 49.29, respectively, whereas the sister group of Fraxininae and Oleinae had higher support values and concordance factors (Fig. 8a and b).

All 15 possible topologies appeared in the 1865 gene trees (Additional file 1: Table S8), and three topologies were the most frequent (> 15%). A total of 30.03% of these gene trees (topo1) were consistent with the species tree. The second and third most frequent topologies (topo2 and topo3, accounting for 18.28% and 17.80% gene trees, respectively) showed Schreberinae as sister to the Fraxininae–Oleinae clade, and forming a clade with Ligustrinae, respectively (Fig. 8d). There was significant branch length variation among the four subtribes of Oleeae (Fig. 8c, one-way analysis of variance test, *P* < 0.05), indicating that heterotachous evolution, such as the rate variation of the lineages, was a likely factor affecting tree discordance. The ASTRAL polytomy test results also rejected the null hypothesis that any branch is a polytomy (*P* < 0.01) in the four subtribes.

*D*-statistics showed no or little gene flow among the four subtribes (Fig. 8e). Gene flow was only identified between Ligustrinae and Oleinae, as well as Ligustrinae and Fraxininae, but the *D* values were much lower than most in the five tribes (Additional file 1: Table S9). QulBL analysis revealed that only one of the six species pairs showed significant evidence for introgression (Fig. 8e, and Additional file 1: Tables S10-S11), suggesting that ILS was the main factor behind gene tree discordance among the four subtribes. PhyloNet analyses supported two reticulation events, between Ligustrinae and the ancestral lineage of Fraxininae and Oleinae, and between Fraxininae and Oleinae (Fig. 8f and Fig. 8g). These two reticulation events were also supported by the *D*-statistic or QulBL.

In summary, our results revealed that ILS and ancient introgression had both contributed to phylogenetic discordance among the four subtribes of tribe Oleeae. Two introgression events were supported: one between Ligustrinae and the ancestral lineage of Fraxininae and Oleinae and the other between Fraxininae and Oleinae.

### Timescale for the Oleaceae tree of life

Using the 91s77G dataset and four calibration priors (Additional file 1: Table S12), we inferred the divergence times of Oleaceae (Additional file 2: Fig. S5). The Oleaceae stem node dated back to the Paleocene (62.59 Ma, 95% highest probability density, HPD: 60.63–64.53 Ma) and the crown node was 60.51 Ma (95%, HPD: 56.01–64.07 Ma). From the late Paleocene (60.51 Ma) to the early Eocene (52.47 Ma), an approximately 8 Ma interval, five ancestral lineages corresponding to the tribes became genealogically divergent. The crown ages of Myxopyreae, Forsythieae, Jasmineae, and Oleeae were dated to 29.47 Ma during the early Oligocene, 19.22 Ma during the early Miocene, 37.78 Ma during the late Eocene, and 46.66 Ma during the middle Eocene, respectively. The four subtribes of Oleeae diverged from 46.66 Ma to 39.43 Ma during the middle Eocene, and the crown ages for the four subtribes were 22.51 Ma, 34.06 Ma, 27.69 Ma, and 33.78 Ma, respectively.

## Discussion

### Variation in substitution rates among the clades of Oleaceae

Our study clearly suggests faster rates of genome evolution in tribe Jasmineae and some branches of the Oleeae subtribe Ligustrinae than in the other clades of Oleaceae, as evidenced by longer branch lengths and larger genetic distances in Jasmineae and Oleeae subtribe Ligustrinae as well as branch model tests. The branch model test in baseml/PAML, e.g., the M1 model (Table 4) shows a 5.5-fold average variation among Jasmineae and the rest of the clades in Oleaceae.

In comparison to previous results, we here report that the lower phylogenetic signal of the deep branching is related to extreme variation in substitution rates in Oleaceae. We sampled representatives of nearly all genera and inferred broad relationships of tribes and subtribes of Oleeae using heterogeneous models (e.g., PMSF, GHOST) and multiple partitioning schemes; however, the deep nodes had low support values and showed conflicts with species trees (Fig. 2 and Additional file 1: Table S3 see below for more details), suggesting that rate heterogeneity severely obscured plastid relationships [43].

Variations in substitution rates among different lineages have long been studied in plants [44–47]. A hypothesis commonly invoked to explain rate variation is generation time, i.e., nucleotide substitution rates are negatively correlated with generation time. This hypothesis has been supported in plants by comparing the rates of long-lived woody plants and short-lived herbaceous plants [44, 45]. Our results also support the generation time hypothesis, as Jasmineae species are woody climbers, shrubs, and herbs, while the remaining Oleaceae species are mostly woody. However, the mechanism behind the influence of generation time on the substitution rate is unclear in plants because different from animals, plants do not sequester their germ line, and somatic mutations can be passed down. Lanfear et al. [48] found a consistently negative relationship between plant height and substitution rate across angiosperms. Differences in the rates of mitosis in the apical meristem can account for the observed differences in rates of molecular evolution among plants of different heights [48]. Taller, long-lived woody plants accumulate more mutations per generation, and the chances of deleterious mutations are increased. A way to avoid this is for them to have fewer opportunities for DNA replication errors to occur than the short-lived plants [49].

Species diversification in angiosperms is positively correlated with substitution rates [49, 50]. In the results of Oleaceae, this correlation is also supported, as Jasmineae is the most species rich (with approximately 220 species throughout the Old World tropics and warm temperate regions) in comparison with the other major clades in the family [27].

Approximately 20% of angiosperm species have biparental plastid inheritance [51, 52], and plastid genome rearrangement events are associated with this inheritance [53–57]. *Jasminum* is a group with biparental plastid inheritance, and the plastid genomes of *Jasminum* and *Menodora* show several distinctive rearrangements, including inversions, gene duplications, insertions, inverted repeat expansions, and gene and intron losses [58]. Meanwhile, the substitution rate is correlated with plastid genome rearrangements [46, 59, 60]. A possible explanation for this is that the biparental inheritance of plastomes influences both substitution rates and plastid genome rearrangements. A scenario may be aberrant DNA repair/recombination/replication (RRR) by biparental inheritance responsible for the increase in substitution rates and highly rearranged plastomes [59, 61].

### Strong discordance among gene trees

The results showed strong discordance of gene trees among different datasets and phylogenomic methods. Exploration of gene tree discordance is fundamental to unravel recalcitrant backbone relationships of Oleaceae, and multiple types (whole plastomes, nuclear SNPs, and multiple nuclear genes) of data were used to tease apart alternative hypotheses concerning the source of gene tree heterogeneity along the backbone phylogeny of Oleaceae.

Although the plastid analyses largely resolved relationships of the olive family, we identified multiple instances of strongly supported conflicts among datasets, sequence types (nucleotide vs. amino acid), and phylogenetic models. In the 19 gene trees based on the plastid datasets, we recovered conflicting or uninformative support at ~ 33% of nodes (Additional file 2: Fig. S2). The sources of conflict in plastid genome phylogenies remain unclear and poorly understood, and several factors have demonstrated their relevance, such as phylogenetic signals, rapid radiation, and rate heterogeneity [6, 62]. In Oleaceae, the rate heterogeneity among the clades likely explains the deep-branching node conflict, and using the amino acid dataset to reduce the observed conflict and rapid radiation may explain the conflict of shallow nodes [35, 37]. Nevertheless, heteroplasmic recombination deserves consideration in light of supported conflict [6].

Our analyses clearly show that the plastid gene tree conflicts with the nuclear SNP gene tree among terminal branches, as well as in some deeper nodes (Fig. 5a). Cytonuclear discordance is well known in plants and has been traditionally attributed to chloroplast capture. Recently, ILS, organellar introgression, positive selection, branch length, and geography have largely explained the widespread cytonuclear discordance in closely related taxa [10, 16, 63]. For the deep nodes, the majority of the incongruences within the olive family can be explained by ancient introgression. For intraspecific or intrageneric relationships, these discordances probably mirror the differences in evolutionary processes (e.g., differences in effective population size and different rates of pollen and seed gene flow) [22, 63]. Nevertheless, allopolyploidization likely explains a portion of the observed discordance. Several species (e.g., *Fraxinus chinensis*, subspecies of *O. europaea*) have been demonstrated to be of recent hybrid origin [29, 64, 65].

Based on the phylogenetic analyses, ancient introgression and ILS were mainly responsible for the phylogenetic discordance observed in the deeper nodes. However, the phylogenetic results had similar phylogenetic information/signals, and it is difficult to differentiate ancient introgression and ILS [66], especially with deep divergence as the earliest dichotomy. Indeed, gene tree discordance caused by ILS is thought to be common when internodes are short owing to rapid diversification [5, 13, 25], and this is often a main factor to explain gene tree discordance at all taxonomic levels. Using the *D*-statistic, QuIBL, and phylogenetic network, we attempted to differentiate the deep coalescence and post-speciation gene glow at the tribe level and the subtribe level, respectively. The *D*-statistic showed the signal of introgression in seven possible locations, and QuIBL was detected in all possible locations among the five tribes of Oleaceae (Fig. 6f). The inferred introgression events agreed with the reticulation scenarios from the phylogenetic network analysis (Fig. 6g–i). The signal of *D*-statistic may be lost or distorted, when there were multiple or “hidden” reticulations [67], was the cause that no introgression was detected between Oleeae and Jasmineae, but it was detected in QuIBL and phylogenetic network analysis. Our phylogenetic tree also exhibited short internal branches at deep branching (Fig. 2), and the distribution of gene tree frequency supports the presence of polytomous topology (Additional file 1: Table S4); however, the polytomy test in ASTRAL rejected a polytomous topology in the five tribes. Indeed, ancient introgression, not ILS, is consistent with our findings and with the extensive discordance we identified in our phylogenetic analyses of the five tribes.

The level of post-speciation gene flow inferred with the *D*-statistic and QuIBL test was very low (Fig. 8e), and ILS was the main cause of the gene tree discordance within the subtribes of Oleeae. Ancient admixture of ancestral lineages is a powerful means for rapid radiation to occur [68]. The results of our phylogenetic analyses, QuIBL tests, and phylogenetic networks support that Oleeae is likely to be the result of ancient allopolyploidization and rapid radiation.

### Early evolutionary history of Oleaceae

**Fig. 9:**
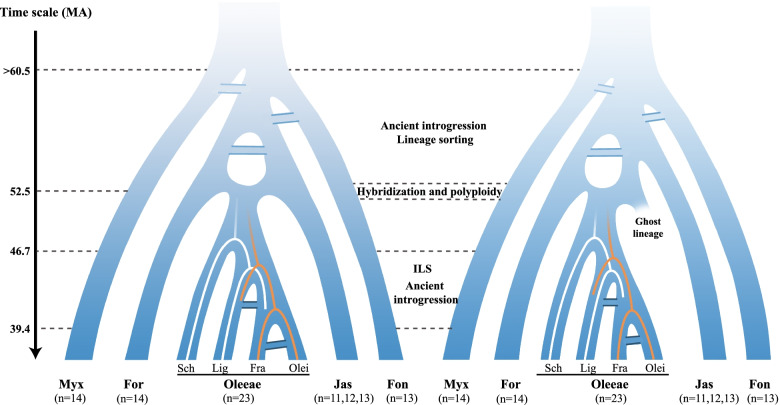
Two alternative models of the evolutionary diversification of Oleaceae. Myx, Myxopyreae; Fon, Fontanesieae; For, Forsythieae; Jas, Jasmineae; Ole, Oleeae; Lig, Ligustrinae; Sch, Schreberinae; Fra, Fraxininae; Olei, Oleinae

We propose two scenarios for the early diversification of Oleaceae based on the results of this study (Fig. 9). The species tree from the nuclear genes and the gene tree from SNPs supported the relationships among the five tribes of the olive family as (Myxopyreae (Fontanesieae, (Forsythieae, (Jasmineae, Oleeae))). Oleaceae originated in the Paleocene, and the first divergence of Myxopyreae from the remaining clades was at c. 60.5 Ma; within approximately eight Ma, five major lineages corresponding to the five tribes became diversified. During these times, there was frequent reticulate evolution. The basic chromosome number [27, 69] and the phylogenomic results [29, this study] support that the tribe Oleeae originated via ancestral allopolyploidization at c. 52.5 Ma. All plastid datasets showed Jasmineae as sister to Oleeae, supporting that the ancestral Jasmineae was the maternal parentage (left scenario in Fig. 9); however, phylogenetic network results did not support the inheritance probability of potential parents (Jasmineae and Forsythieae) of approximately 50%, also consistent with low-level gene flow using the *D*-statistic and QuIBL test (Fig. 6f–i). Moreover, the results from genome synteny analyses revealed both *O. europensa* and *Fraxinus excelsior* of tribe Oleeae showed higher genome synteny to tribe Forsythieae (*Forthysia suspensa*) than to tribe Jasmineae (*J. sambac*), indicating the ancestral lineages of Jasmineae may not be the direct ancestors (Fig. 7). We hence propose an alternative scenario in which there was a “ghost lineage,” which was sister to Jasmineae, and this extinct “ghost lineage” was the likely maternal parent of the tribe Oleeae. Phylogenetic network analysis strongly support that the ancestral Forsythieae was the paternal parentage. The allopolyploid Oleeae experienced a rapid radiation, and the most likely species tree of the four subtribes is (Schreberinae, (Ligustrinae, (Fraxininae, Oleinae))). ILS, together with the limited introgression, is the most likely driving force for the divergences of the four subtribes of Oleeae.

## Conclusions

In this study, we employed multiple genomic datasets to resolve the phylogenetic relationships, especially the deep nodes of the olive family Oleaceae. Analyses of the whole plastid genome and the nuclear genes provide evidence for extreme heterogeneity of plastid substitution rates among the different clades, and these findings have implications for systematics of the family. Although our phylogenetic results confirm support for monophyly of the family and each of the five tribes and the four subtribes of tribe Oleeae, we have also detected strong conflicts in relationships inferred from the plastid and nuclear SNP datasets, as well as the nuclear gene trees. By evaluating conflicting phylogenetic signals, we have resolved the backbone phylogeny of Oleaceae and have detected ancient introgression and ILS in the deeper nodes. More generally, this study adds valuable genomic data of the economically important olive plant family and explores gene tree discordance in detail, providing a strong case study on exploring the complexity of the plant tree of life in the genomic age.

## Methods

### Taxon sampling, plant material, and the deposition of vouchers

We sampled 179 ingroup samples, including 140 species and one outgroup (Carlemanniaceae, *Carlemannia griffithii*), which was the sister family of Oleaceae. The ingroup included species representing all currently recognized tribes (five), subtribes (four), and genera (24) (except the genus *Dimetra*, which only included one species, *Dimetra craibiana*), in Oleaceae according to the classifications of E Wallander and VA Albert [26] and PS Green [27]. Eighty-four samples were obtained in this study (Additional file 1: Table S1), and 96 samples were from GenBank (Additional file 1: Table S2).

The 84 samples obtained in this study were mainly collected from the field and herbarium specimens. All samples were identified based on morphological characters. Leaf material from the field was dried using silica gel, and the voucher specimens were deposited in the herbarium of the Institute of Botany, Chinese Academy of Science (PE). The herbarium materials were obtained from PE, and the specimens were selected using the two criteria according to the results of Xu et al. [70]: (1) the collection date for the specimen was as close to today as possible and (2) the specimen was from a healthy plant. Every specimen was inspected under a dissecting microscope to ensure that there were no visible fungal infections. All the samples were collected according to the local, national, or international guidelines and legislation.

### DNA isolation and sequencing

Leaf material was ground using the mechanical lapping method, and the total DNA was isolated using a modified CTAB protocol (mCTAB) [71]. DNA concentration was measured with the Qubit 2.0 Fluorometer (Thermo Fisher Scientific), and the length of the DNA fragments was quantified on an agarose gel for a subset of the samples. Total DNA concentrations > 1 μg were chosen for Illumina sequencing.

Genome skimming was used to obtain plastid genome data and nuclear SNPs and to identify multiple nuclear genes [35, 72]. Total DNA was fragmented by sonication into 350 bp fragments except for some herbarium materials that had degraded to less than 350 bp. The DNA was constructed as 350-bp insert libraries, and the degradation DNA of herbarium material was used to construct 200-bp insert libraries using Nextera XT DNA Library Preparation Kit (Illumina, San Diego, CA, USA) and was then used for sequencing. Each sample was paired-end sequenced (150 bp) on the Illumina HiSeq X-ten at Novogene in Tianjin, China. Most samples yielded approximately 5 Gb of 150-bp paired-end reads. The samples were used to sequence whole genomes, yielding 35 Gb of data.

### Plastome assembly and annotation

Raw reads were cleaned and filtered as follows: Illumina adapter artifacts, low-quality reads and low-quality bases at the read ends were trimmed with Trimmomatic 0.39 (using settings: ILLUMINACLIP:TruSeq3-PE.fa:2:30:10:1:true LEADING:20 TRAILING:20 SLIDINGWINDOW:4:15) [73]. Two methods were used to assemble the plastomes. First, the whole plastomes were assembled using GetOrganelle [74]. with a range of k-mers of 65, 75, 85, 95, and 105. If GetOrganelle was unsuccessful at assembling complete plastomes, we used the second method to assemble it.

For the second successive assembly method, clean data from Trimmomatic were assembled *de novo* into contigs using SPAdes version 3.13.1 [75]. The plastome contigs were extracted directly by BLAST search from the de novo assembled contigs against *Fraxinus excelsior*, *Jasminum nudiflorum*, and *Olea europaea* plastome reference sequences using custom Python scripts. The extracted contigs were further assembled using Sequencher v5.4.5 (Gene Code Corporation, Ann Arbor, MI, USA). The gaps between the contigs were filled using clean reads that were mapped to the contigs. The plastomes were further checked by mapping the paired reads to the assembled plastomes and scanned by eye to confirm appropriate mapping using Geneious Prime version 2020.0.5 [76].

Finished plastomes were annotated using the Perl script Plann [77], and the missing or incorrect genes were checked in Geneious. The physical maps of the Oleaceae were drawn using OrganellarGenomeDRAW [78]. Finally, the newly assembled plastomes and the raw Illumina data were deposited in GenBank (Additional file 1: Table S1).

### Nuclear SNP calling

Olofsson et al. [35] described a reference-based approach to call SNPs using low-depth whole genome sequencing data. This method used the quality filtered reads to map onto a reference genome and extracted the high-quality SNP positions from uniquely mapped reads taking differences in sequencing depth between samples into account [35] and then bioinformatically reconstructing genotypes from uniquely mapped reads using a series of bioinformatic pipelines. Three whole genomes of Oleaceae were used as the reference genomes for SNP calling. The oleaster (*Olea europaea* var. *sylvestris*) [79] and ash (*Fraxinus excelsior*) [80] both belong to tribe Oleeae, and *Forsythia suspensa* [81] belongs to tribe Forsythieae.

Raw reads were first subjected to quality control using the NGS QC toolkit version 2.3.3 [82]. Reads with more than 20% of bases with quality scores below 20 were removed, and low-quality bases (Q < 20) were trimmed from the 3′ end of each read. Quality-controlled reads of all 180 samples were mapped to the four reference genomes using Bowtie 2 [83], and uniquely mapped reads in proper pairs were identified using SAMtools version 1.3.1 [84] and Picard tools version 1.92 (http://broadinstitute.github.io/picard/). The high-quality nuclear SNPs were called in SAMtools [84] using the “mpileup” module. The individual genotypes were merged in BCFtools version 1.3.1 [85] filtered in VCFtools version 0.1.14 according to the following criteria: (1) quality value ≥ 20; (2) for each sample, the raw genotyped SNPs were filtered, and the sites with coverage between 0.5 and two times the median coverage; (3) a minor allele count of at least three; and (4) SNPs with ≥ 20 missing genotypes within the 180 samples were removed.

### Plastid gene/genome alignment and data matrix construction

#### Whole plastid genome datasets

In total, 180 whole plastomes were aligned (excluding one copy of the inverted repeat) using Mauve Version 1.1.1 [86] to identify potential genome rearrangements such as inversions. The genome rearrangements were adjusted manually according to the gene order of *Fraxinus excelsior*. The alignment was done using MAFFT version 7.313. As regions of introns and spacers can be difficult to align at high taxonomic levels, we used TrimAl version 1.3 [87] to explore the effect of inferring phylogenetic relationships based on the four automated trimming methods (Table 1).

#### Protein coding loci

GenBank files were generated in Sequin for all the newly assembled plastomes, and other Oleaceae plastome data were downloaded from GenBank. The coding genes were extracted from the annotated plastomes using a custom Python script. Each gene was aligned with the codon-based alignment model in the MAFFT version 7.313 plugin in PhyloSuite version 1.2.2 [88]. The *ycf1* and *ycf2* genes were excluded from the following analyses because of the greater number of indels in the alignment. Alignments were visualized and concatenated in PhyloSuite version 1.2.2. The resulting matrix comprised 77 protein-coding genes, 180 samples, and 55,296 aligned bp.

Three separate protein-coding matrices were analyzed: (1) “180s77Gnt,” the nucleotide sequences of all protein coding loci including all taxa; (2) “180s77Gaa,” the amino acid sequences of all protein coding loci including all taxa; (3) “91s77G,” a reduce sample set from 180s77Gnt with nearly all representative lineages of Oleaceae used for divergence time analyses.

### Orthologous nuclear gene identification

Eight species from Oleaceae (one species represented each tribe or subtribe) and *Origanum vulgare* from Lamiaceae were used to identify orthologous gene families. Four species (Myxopyreae: *Myxopyrum hainanense*, Fontanesieae: *Fontanesia phillyreoides*, Jasmineae: *Jasminum mesnyi*, and Oleeae subtribe Ligustrinae: *Syringa pubescens*) were subjected to whole genome sequencing, and the sequencing depth was approximately 30X. The raw data of *Schrebera swietenioides* (Oleeae subtribe Schreberinae) were downloaded from the SRA database (SRR8247314). Three sequenced genomes of Oleaceae plants, including *Fraxinus excelsior* (Oleeae subtribe Fraxininae), and *Olea europaea* (Oleeae subtribe Oleinae), *Forsythia suspensa* (Forsythieae), and the outgroup *Origanum vulgare* (Lamiaceae), were downloaded from the published database.

The raw data were subjected to Trimmomatic 0.39 for quality control and assembled de novo into contigs using SPAdes 3.6.1 [75]. The completeness of the assembled genome was estimated by BUSCO 4.0 [89]. Groups of orthologous sequences were defined using OrthoFinder2 [90] under the parameters *S* = diamond. Each single-copy orthogroup was aligned via MAFFT version 7 [91] with the setting “--auto,” and all alignments were further trimmed using TrimAl version 1.2 [87] with the “automate1” method.

To reveal the evolutionary history of Oleaceae at different levels, two nuclear datasets were constructed at the tribe and subtribe levels. The tribe nuclear dataset included five ingroups (one species representing each tribe, i.e., *Myxopyrum hainanense*, *Fontanesia phillyreoides*, *Forsythia suspensa*, *Jasminum mesnyi*, and *Fraxinus excelsior*) and one outgroup species (*Origanum vulgare*). A total of 2,608 single-copy orthologous genes, which were more than 300 bp in length, were identified. The nuclear dataset of subtribe Oleeae includes four ingroups (one species representing each subtribe, i.e., *Schrebera swietenioides*, *Syringa pubescens*, *Fraxinus excelsior*, and *Olea europaea*) and one species of *Forsythia suspensa*. A total of 1865 single-copy orthologous genes were identified using OrthoFinder2.

### Gene tree reconstruction based on plastid and SNP datasets

Gene trees were reconstructed using the maximum likelihood (ML) methods as implemented in the programs RAxML-NG [92] and IQ-TREE 2 [93]. RAxML-NG is a from-scratch reimplementation of the established greedy tree search algorithm of RAxML/ExaML, and it offers improved accuracy and speed [92]. IQ-TREE is a user-friendly and widely used software package for phylogenetic inference using maximum likelihood and supports more evolutionary models.

Each analysis used the best fit models, which were selected using ModelFinder [94]. For the datasets 180s77Gnt and 180s77Gaa, we used the following partition schemes: (i) unpartitioned, (ii) partitioned according to results from PartitionFinder 2 [95] with predefined partitioning by genes, (iii) partitioned by genes, and (iv) partitioned by codons (only in 77G180snt dataset). All partitioning analyses were run in PartitionFinder 2 [95] under the model selection Akaike Information Criterion criteria (AICc) and with branch length linked. RAxML-NG [92] was run for the ML tree with 500 bootstrap replicates. In order to investigate phylogenetic incongruence within the SNP data, we used the dividing method, thereby avoiding to simply include concatenation-based ML analyses based on the GTR+G model. The SNP-ash dataset was used for this analysis, because of this dataset included the most number of SNPs. Each 10 kb of the SNPs were divided into a new data matrix and used for tree reconstruction.

Many studies have shown that heterotachous evolution, i.e., rate variation across sites and lineages, may mislead phylogenetic inference [11, 96, 97]. The posterior mean site frequency (PMSF) model [98] and general heterogeneous evolution on a single topology (GHOST) model [99] were used to reconstruct alternative trees. The PMSF model implemented in IQ-TREE considers mixture classes of rates and substitution models (here, the LG model) across sites as a rapid approximation to the CAT model in PhyloBayes [100]. The dataset 180s77Gaa was used for PMSF phylogenetic reconstruction because this method only supported the amino acid data. Specifically, we used the LG + C60+G+F model for PMSF phylogenetic reconstruction. PMSF requires a guide tree, which we obtained from RAxML-NG analysis. Nodal support was assessed with 1000 replicates of the ultrafast bootstrapping (UFBoot) method [101].

GHOST is an edge-unlinked mixture model consisting of several site classes, each having a separate set of model parameters and edge lengths on the same tree topology. All nucleotide datasets were used to infer phylogenetic relationships using this model implemented in IQ-TREE. Branch support values were computed using the UFBoot method.

### Comparison of multiple trees

The normalized Robinson-Fould’s distance (RF) was used to examine the topological congruence between each gene tree. The RF distance was calculated using IQ-TREE. Principal coordinates analysis (PCoA) based on the RF distance was used to assess the clustering pattern of multiple trees, which calculates the best reduced-spaced visualization of the distances between trees. PCoA performed using R.

Concordance among the trees generated from the plastid datasets and SNP datasets was analyzed using PhyParts [102] and visualized using PhyParts_PieCharts (https://github.com/mossmatters/MJPythonNotebooks; last accessed August 13, 2021). Both internode certainty all (ICA) values and conflicting/concordant bipartitions were calculated. For these analyses, branch support values less than 80% were cut off, and this node was regarded as uninformative for the reference tree node.

### Assessment of discordance between gene trees and the species tree

For the nuclear single-copy orthologs, we used RAxML-NG to infer the best ML trees from unpartitioned alignments for each locus using a GTR + G substitution model, and the branch support value was computed with 200 bootstrap replicates.

Species trees were reconstructed by summarizing gene trees using ASTRAL-III [42]. Local posterior probabilities (LPPs) were calculated for branch support [103]. We further used the quartet scores (QS), gene concordance factor (gCF), and site concordance factor (sCF) to measure the amount of gene tree conflict around each branch of the species tree. The QS was calculated in ASTRAL to examine the number of gene tree quartets supporting the primary (q1), second (q2), and third (q3) alternative topologies. gCF and sCF represent the percentage of decisive gene trees and sites supporting a branch in the reference trees [104], respectively. gCF and sCF were computed in IQ-TREE.

To further visualize conflict, we built a density tree from 500 gene trees randomly sampled using the Toytree Python toolkit (https://github.com/eaton-lab/toytree; last accessed August 13, 2021). All gene trees were converted to ultrametric trees in TreePL [105].

We also used topological weighting to reduce the complexity of the six-taxon phylogeny of the Oleaceae and the five-taxon phylogeny of the tribe of Oleeae. Ignoring the branch length, there are 105 and 15 types of topologies within a rooted binary tree of six and five terminal branches. We calculated the frequency of the alternative topologies using the Python script (twisst.py; https://github.com/simonhmartin/twisst; last accessed August 13, 2021).

### D-statistic

We analyzed the *D-*statistic in the form *D* = (nABBA-nBABA)/(nABBA+nBABA) in a rooted tree (((P1, P2), P3), O) to assess whether species P1 or P2 had gene flow with P3. The null hypothesis about no gene flow between the species is rejected when the *D*-statistic significantly deviates from 0 [106, 107]. We used a threshold *Z* > 3 to reject the null hypothesis, which corresponds to *P* < 0.002. In the outcome of the *D-*statistic analysis, P2 and P3 had gene flow if a *Z*-score > 3 and a *D*-score > 0, and P1 and P3 had gene flow if a *Z*-score > 3 and a *D*-score < 0. All possible combinations of the four-taxon topology were subjected to the *D*-statistic analyses using the evobiR package in R (https://github.com/coleoguy/evobir; last accessed August 13, 2021).

### QuIBL

QuIBL is based on the analysis of branch length distributions across gene trees to infer putative introgression patterns, which can be used to test hypotheses of whether phylogenetic discordance between all possible triplets is explained by ILS alone or by a combination of ILS and gene flow [19]. QuIBL uses the distribution of internal branch lengths and calculates the likelihood that the discordant gene tree is due to introgression rather than ILS. The Bayesian information criterion (BIC) was used to test whether the gene trees discordant from the species tree were more similar to introgression or ILS. We used a stringent cutoff of dBIC < − 10 to accept the ILS + introgression model, as suggested by the author [19]. The single-copy orthologous genes were used for QuIBL analyses.

### Species network analysis

We inferred a species network to assess the effect of gene tree conflicts due to hybridizations. A species network based on the gene trees from the single-copy orthologous genes was carried out using the maximum pseudolikelihood method InferNetwork_MPL included in the package PhyloNet [108]. We carried out three network searches by allowing one to three reticulations and performed 10 independent searches for each reticulation setting to avoid local optima. The optimal networks were displayed in Dendroscope 3 [109].

### Polytomy test

To test whether the gene tree discordance could be explained by polytomies instead of bifurcating nodes, quartet-based polytomy tests were carried out in ASTRAL-III following Sayyari and Mirarab [110]. Quartet frequencies for all branches were inferred using the gene trees to determine the presence of polytomies, where *P* < 0.05 was considered to reject the null hypothesis of a polytomy. The analysis was run second to minimize error due to gene tree error (collapsing branches with < 50% bootstrap support).

### Genome synteny analysis

We downloaded four genomes: *Forsythin suspensa* (Accession Number: GCA_020510225.1) of tribe Forsythieae [111], *Jasmimum sambac* (Accession Number: GCA_018223645.1) of tribe Jasmineae [112], and *Olea europaea* (Accession Number: GCA_002742605) and *Fraxinus excelsior* (Accession Number: GCA_019097785) of tribe Oleeae [79, 113]. Transcripts of *O. europaea* and *F. excelsior* were downloaded as well. We first ran BLAST search of transcript of *O. europaea* against genomes of *F. suspensa* and *J. sambac*, respectively. We used whole transcripts of *O. europaea* and *Fraxinus excelsior* separately as cut-offs for BLAST matches, max e-value was set to 1e^−5^ during the analysis. When one cut-off matched to multiple locations, we retained the match with the highest hit-score and removed the rest to ensure that one cut-off matched to only one position on the genome.

We compared genome synteny among *O. europaea*, *J. sambac*, and *F. suspensa*, based on the results from the BLAST search. Genome synteny between *F. excelsior* and the putative parental lineages was analyzed with the same method. Local BLAST database construction and BLAST search were run by Geneious Prime [76], while genome synteny plots were constructed following the MCscan pipeline from Tang et al [114].

### Time calibration of the phylogeny

We used BEAST v2.5.1 [115] to estimate the divergence times of Oleaceae using the 91s77G dataset. Four calibration priors were utilized in this study (Additional file 1: Table S12). According to the results of Zhang et al. [4], the average age of the most recent common ancestor (TMRCA) of the Oleaceae and Carlemanniaceae (the root of the tree) was 62.23 Ma. The samaras of *Fraxinus wilcoxiana* Berry were described from the Middle Eocene Claiborne Formation of western Tennessee, USA [116]. Following Besnard et al. [39] and Hong-Wa and Besnard [33], we implemented this age as a lower bound of the TMRCA of subtribe Fraxininae and subtribe Oleinae. These fossil priors were given a lognormal distribution with offset values of 40 Ma and a standard deviation of 3 Ma. Fossils of *Olea* subgenus *Olea* occurred before 23 Ma [117–119] and were used to calibrate the crown of *Olea* subgenus *Olea* > 23 Ma. A pollen of *Fraxinus praedicta* Heer from the upper Miocene in Europe (12 Ma) representing the extant taxon *Fraxinus angustifolia* was used to set the minimum age for the living European ashes (set to the crown of *F. angustifolia* and *F. excelsior*) [117]. For these two priors, we used lognormal distributions with offset values of 23 and 12 Ma, respectively, and a mean of 1 Ma and a standard deviation of 0.5 Ma, allowing for the possibility that these nodes are considerably older than the fossils themselves.

We ran analyses with the GTR + G site model, relaxed clock lognormal to account for rate variability among lineages, Yule tree speciation models, and 500,000,000 generations with the MCMC method. The sampling frequency was 50,000 generations, and the adequacy of the parameters was checked using Tracer 1.6 [120] to evaluate convergence and to ensure a sufficient and effective sample size (ESS) surpassing 200. A maximum clade credibility tree was computed after discarding 10% of the saved trees as burn-in using TreeAnnotator v2.4.7.

### Plastid substitution rate analyses and inference of rate changes

To assess variation in substitution rates among clades among the Oleaceae, node-to-tip branch lengths from the rooted species of each sample were calculated for the ML tree of 180s77gnt based on the gene partition model. Branch lengths were counted using the Toytree Python toolkit. The genetic P-distances between the *Carlemannia griffithii* (the outgroup species) and Oleaceae samples were calculated using MEGA 7.0 [121]. The *t* test was performed using R to test differences in branch lengths and genetic distance among clades.

We used the baseml module of PAML v.4.8 [122] to test the null hypothesis that Oleaceae evolve via a “Global Clock” (all rates equal among the clades/branches). The different “branch models” were tested, allowing rates to vary in prespecified regions of the tree corresponding to clades, as opposed to a “background” rate. Four models were used to test different rates among the clades (tribe or subtribe) in Oleaceae. Model M0 specified a global clock for all Oleaceae; Model M1 allowed Jasmineae to evolve via a local chock; Model M2 allowed local clocks for Jasmineae and Oleeae subtribe Ligustrinae; and Model M3 allowed the four clades of Jasmineae, Oleeae subtribe Ligustrinae, Oleeae, and Forsythieae to have independent local clocks. To evaluate significant differences in model fit, we used likelihood ratio tests and corrected Akaike information criterion comparisons following the method of Barrett et al. [123].

## Supplementary Information

**Additional file 1: Table S1.** Taxa included in this study with locality and voucher numbers. **Table S2.** Information from the GenBank data, including the accession number of chloroplast genome sequences and Sequence Read Archive (SRA). **Table S3.** Branch support values of the 25 gene trees at the tribe level. The number of the trees the same as in Table 2. **Table S4.** Frequency of all the possible tree topologies from six species at the tribe level of Oleaceae. **Table S5.** *D*-statistic test results at the tribe level of Oleaceae with *Origanum vulgare* as an outgroup. **Table S6.** QuIBL analysis results at the tribe level of Oleaceae. **Table S7.** Average total introgression proportion per species pair in the QuIBL analysis at the tribe level of Oleaceae. **Table S8.** Frequency of all the possible tree topologies from five species at the subtribe level of tribe Oleeae. **Table S9.** *D*-statistic test results at the subtribe level of tribe Oleeae with *Forsythia suspensa* as an outgroup. **Table S10.** The QuIBL analysis results at the subtribe level of tribe Oleeae. **Table S11.** Average total introgression proportion per species pair in the QuIBL analysis at the subtribe level of tribe Oleeae. **Table S12.** Details of the four calibrations points used in the BEAST analysis.

**Additional file 2: Fig. S1.** The maximum likelihood tree estimated from the 77G180saa based on the gene partition models used as a reference to evaluate conflict and concordance among the 19 plastid datasets trees (Table 2). Pie charts depict conflict amongst the input trees, with the blue, green, red, and gray slices representing, respectively, the proportion of input bipartitions concordant, conflicting (supporting a single main alternative topology), conflicting (supporting various alternative topologies), and uninformative (BS < 80) at each node. The numbers below each branch are ICA values. **Fig. S2.** The maximum likelihood tree estimated from the SNP-ash dataset used as a reference to evaluate conflict and concordance among the six SNP gene trees (Table 2). Pie charts depict conflict amongst the input trees, with the blue, green, red, and gray slices representing, respectively, the proportion of input bipartitions concordant, conflicting (supporting a single main alternative topology), conflicting (supporting various alternative topologies), and uninformative (BS < 80) at each node. The numbers below each branch are ICA values. **Fig. S3.** The maximum likelihood tree estimated from the SNP-ash dataset used as a reference to evaluate conflict and concordance among the 41 gene trees using the dividing methods. Pie charts depict conflict amongst the input trees, with the blue, green, red, and gray slices representing, respectively, the proportion of input bipartitions concordant, conflicting (supporting a single main alternative topology), conflicting (supporting various alternative topologies), and uninformative (BS < 80) at each node. The numbers below each branch are ICA values. **Fig. S4.** The maximum likelihood tree estimated from the 77G180saa based on the gene partition models used as a reference to evaluate conflict and concordance among the 24 trees (plastid datasets and SNP datasets, Table 2). Pie charts depict conflict amongst the input trees, with the blue, green, red, and gray slices representing, respectively, the proportion of input bipartitions concordant, conflicting (supporting a single main alternative topology), conflicting (supporting various alternative topologies), and uninformative (BS < 80) at each node. The numbers below each branch are ICA values. **Fig. S5.** The divergence time of Oleaceae was estimated by BEAST according to age calibrations of four nodes based on the concatenated 76-coding gene dataset.

**Additional file 3:.** Note. The reason for using the ML tree from the 180s77Gaa dataset under a gene partitioning scheme as the reference tree.

## Data Availability

Illumina sequence reads generated in this study have been deposited at NCBI’s short sequence read archive (SRA) under accession number PRJNA820313 [124] and PRJNA704245 [125]. The samples and the voucher specimens used in this study are deposited at the PE herbarium. Information on the samples can be found in Additional file 1: Table S1.
