# Supplementary figures and images for: Phylogenomic approaches untangle early divergences and complex diversifications of the olive plant family
Source: BMC Biol. 2022 Apr 25;20:92. doi: 10.1186/s12915-022-01297-0 (PMC9040247; doi:10.1186/s12915-022-01297-0)

Figure S1

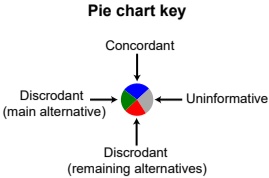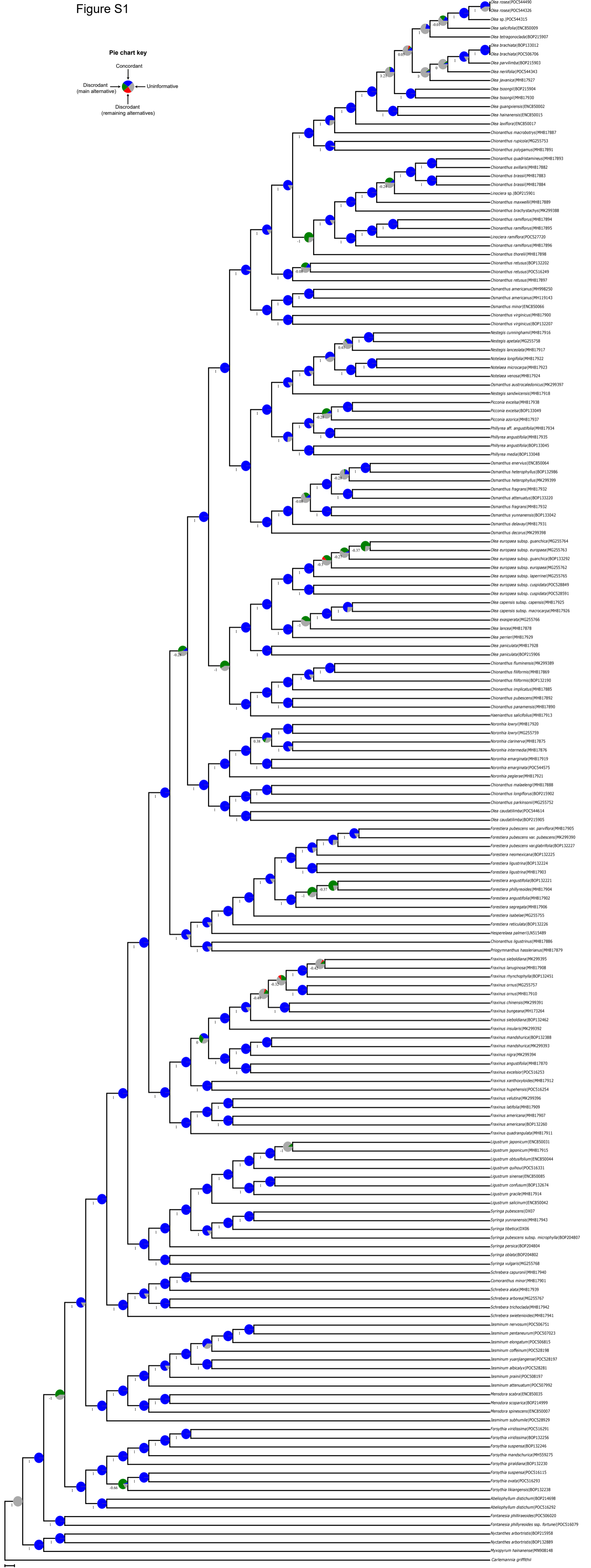

Figure S2

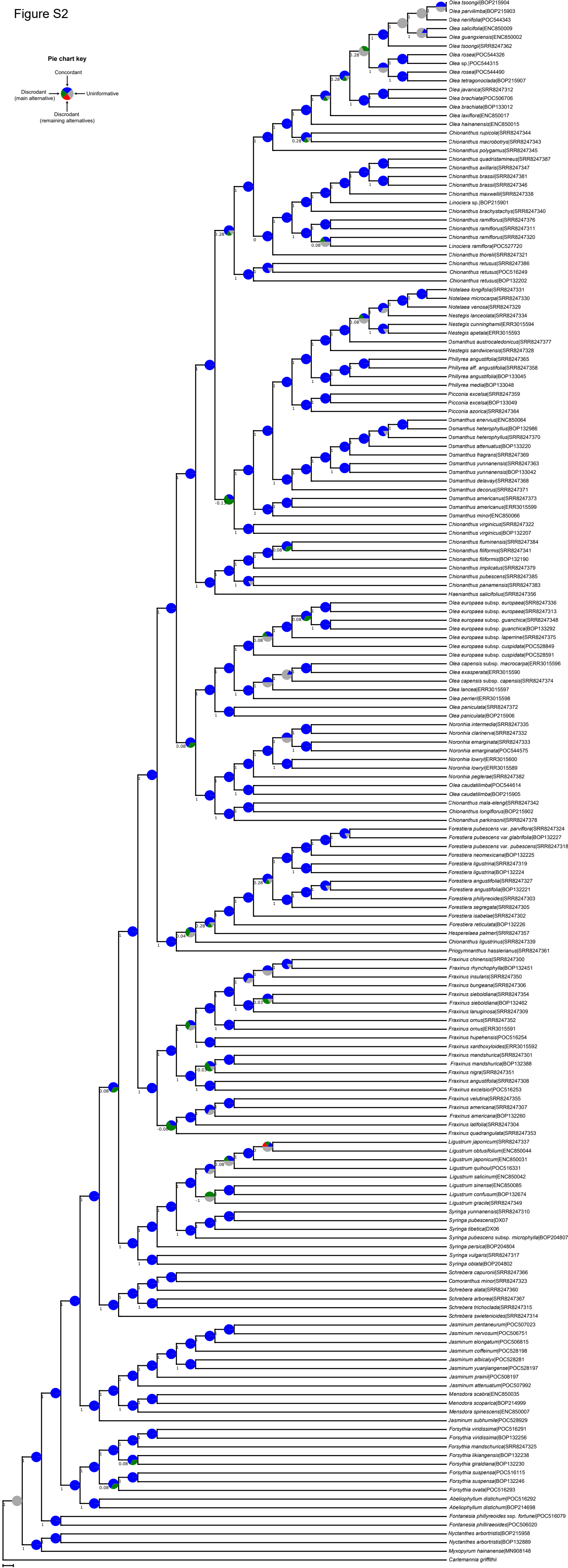

Figure S3

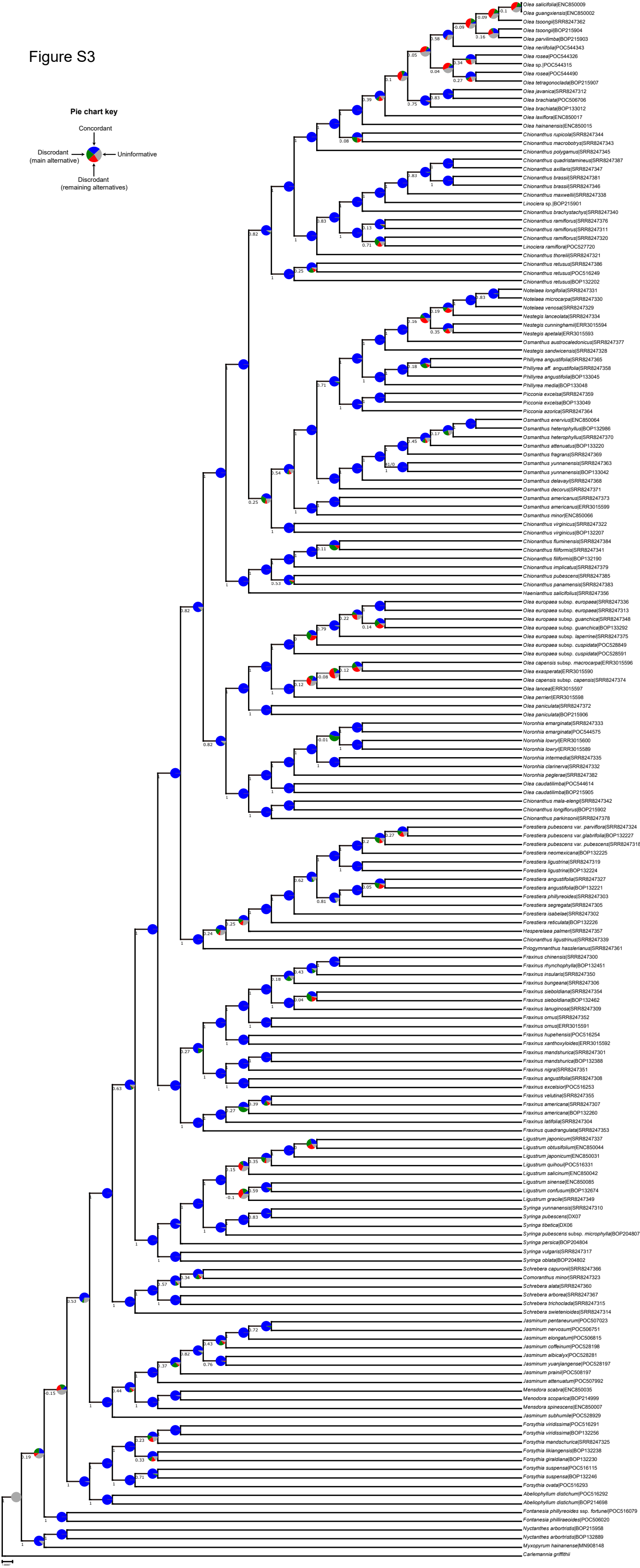

Figure S4

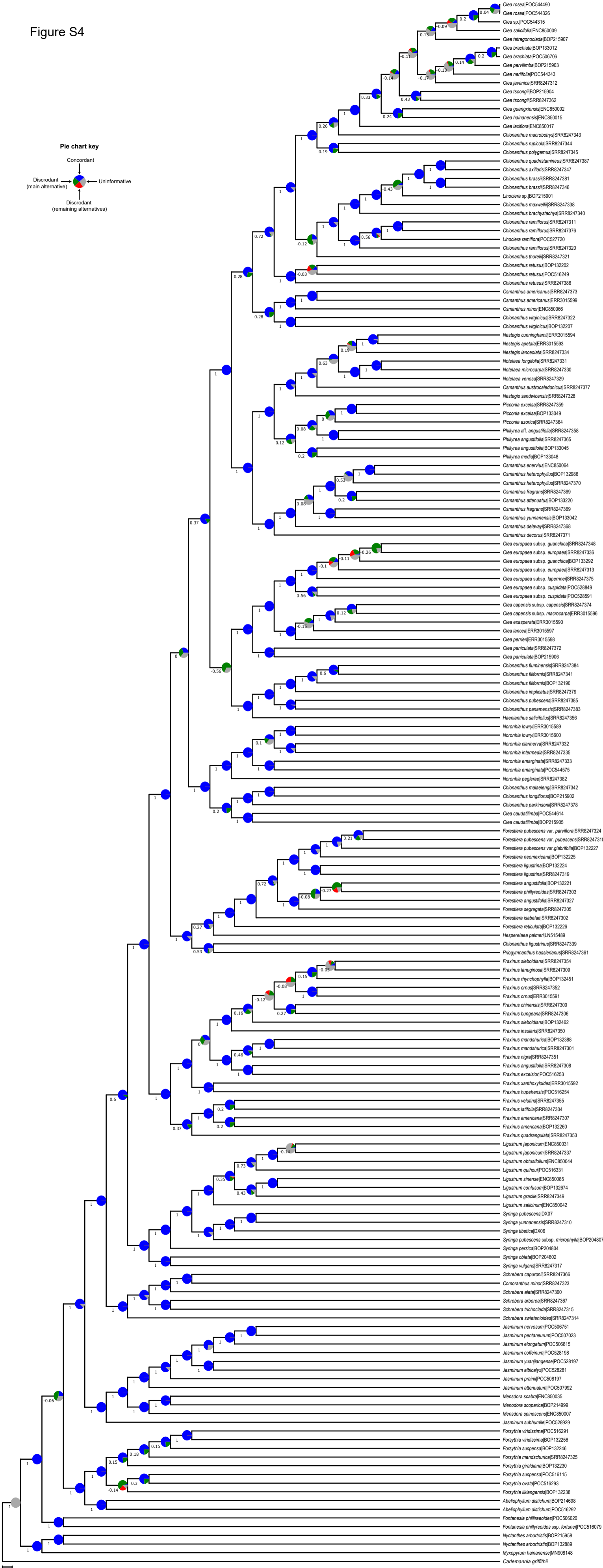

Figure S5

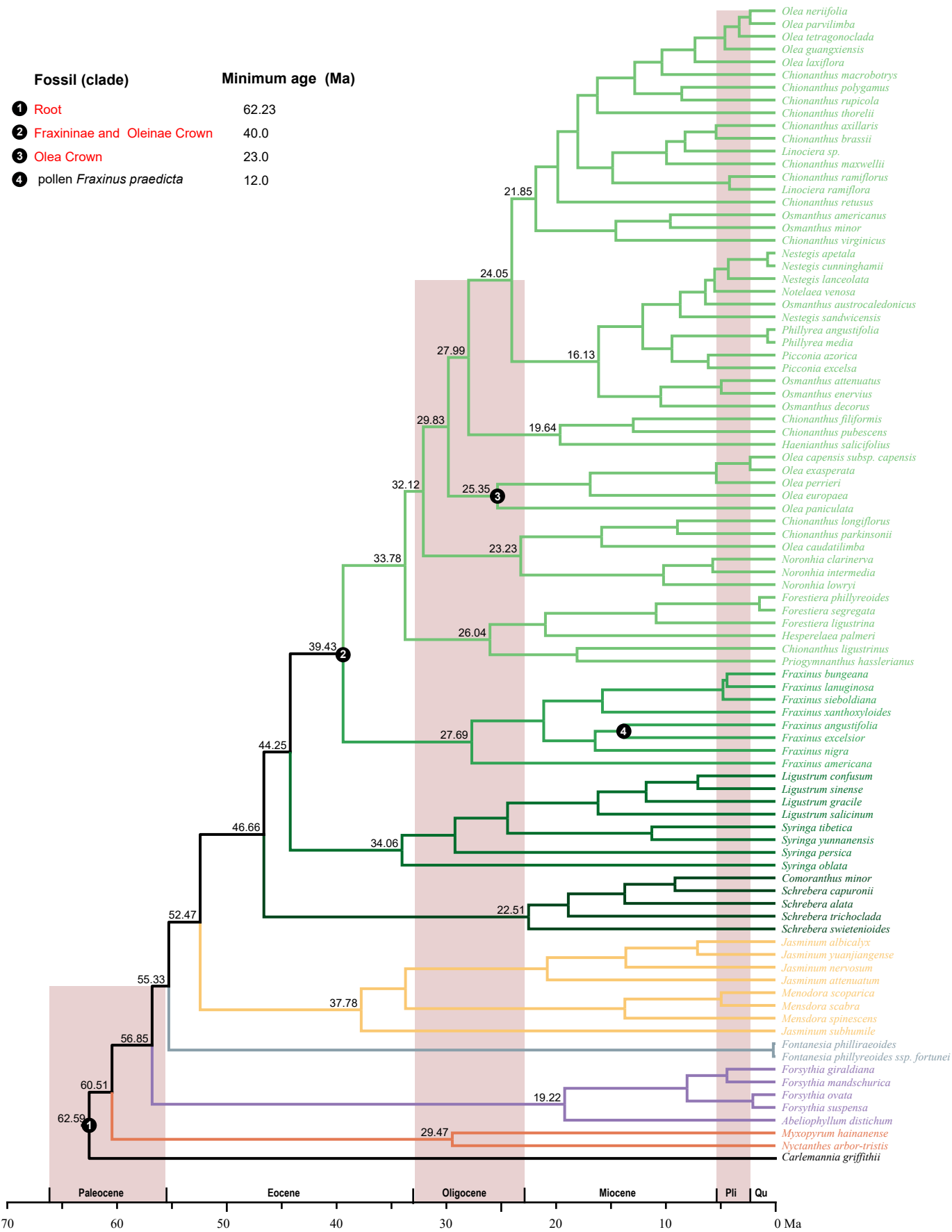

Supplement: Supplementary file 2 — Additional file 2: Fig. S1. The maximum likelihood tree estimated from the 77G180saa based on the gene partition models used as a reference to evaluate conflict and concordance among the 19 plastid datasets trees (Table 2). Pie charts depict conflict amongst the input trees, with the blue, green, red, and gray slices representing, respectively, the proportion of input bipartitions concordant, conflicting (supporting a single main alternative topology), conflicting (supporting various alternative topologies), and uninformative (BS < 80) at each node. The numbers below each branch are ICA values. Fig. S2. The maximum likelihood tree estimated from the SNP-ash dataset used as a reference to evaluate conflict and concordance among the six SNP gene trees (Table 2). Pie charts depict conflict amongst the input trees, with the blue, green, red, and gray slices representing, respectively, the proportion of input bipartitions concordant, conflicting (supporting a single main alternative topology), conflicting (supporting various alternative topologies), and uninformative (BS < 80) at each node. The numbers below each branch are ICA values. Fig. S3. The maximum likelihood tree estimated from the SNP-ash dataset used as a reference to evaluate conflict and concordance among the 41 gene trees using the dividing methods. Pie charts depict conflict amongst the input trees, with the blue, green, red, and gray slices representing, respectively, the proportion of input bipartitions concordant, conflicting (supporting a single main alternative topology), conflicting (supporting various alternative topologies), and uninformative (BS < 80) at each node. The numbers below each branch are ICA values. Fig. S4. The maximum likelihood tree estimated from the 77G180saa based on the gene partition models used as a reference to evaluate conflict and concordance among the 24 trees (plastid datasets and SNP datasets, Table 2). Pie charts depict conflict amongst the input trees, with the blue, gr [file 12915_2022_1297_MOESM2_ESM.pdf]
