# Supplementary material for: Phylogenomic approaches untangle early divergences and complex diversifications of the olive plant family
Source: BMC Biol. 2022 Apr 25;20:92. doi: 10.1186/s12915-022-01297-0 (PMC9040247; doi:10.1186/s12915-022-01297-0)
Supplement: Supplementary file 3 — Additional file 3:. Note. The reason for using the ML tree from the 180s77Gaa dataset under a gene partitioning scheme as the reference tree. [file 12915_2022_1297_MOESM3_ESM.docx]

Two reasons were considered using the phylogeny of 180s77Gaa dataset. First, this tree topology of tribe level was consistent with nuclear genes, but conflicted with other chloroplast genome data. Using this tree as the reference can illustrate the differences clearly among the gene trees. Second, the chloroplast genome showed high rate variation among clades of Oleaceae. The aa dataset reduces the effect of heterotachous evolution, which may mislead phylogenetic inference. This tree obtained from 180s77Gaa dataset thus may be the most “correct” tree based on the chloroplast genome dataset.
